# Cardiac structure and function in patients with inflammatory bowel disease

**DOI:** 10.1016/j.isci.2026.116524

**Published:** 2026-06-27

**Authors:** Yang Yang, Xingyang Luo, Linxiang Zhou, Guanzhong Shi, Ruozhu Yang, Ying Xiao, Xiaowei Liu

**Affiliations:** 1 Department of Gastroenterology, Xiangya Hospital, Central South University, Changsha, Hunan, China; 2 Hunan International Scientific and Technological Cooperation Base of Artificial Intelligence Computer Aided Diagnosis and Treatment for Digestive Disease, Changsha, Hunan, China; 3 National Clinical Research Center for Geriatric Disorders, Xiangya Hospital, Central South University, Changsha, Hunan, China; 4 Xiangya School of Medicine, Central South University, Changsha, Hunan, China; 5 Centre for Human Genetics, Roosevelt Drive, University of Oxford, Oxford, UK

**Keywords:** Cardiovascular medicine, Health sciences, Internal medicine, Medical specialty, Medicine

## Abstract

Inflammatory bowel disease is associated with increased cardiovascular risk, motivating a quantitative assessment of cardiac structure and function in affected patients. We conducted a systematic review and meta-analysis of 28 studies including 1613 patients with inflammatory bowel disease and 1303 healthy controls. Compared with healthy controls, patients showed greater posterior wall thickness (standardized mean difference 0.23, 95% confidence interval 0.12–0.34) and interventricular septum thickness (0.22, 0.05 to 0.39), lower ejection fraction (−0.28, −0.41 to −0.14), and less negative global longitudinal strain (0.36, 0.01 to 0.71). These findings suggest modest differences across cardiac structural and functional domains, although individual parameter-level results require cautious interpretation. Exploratory subgroup analyses showed variable patterns by disease activity, age of onset, and disease subtype. Standardized prospective studies are needed to clarify cardiovascular assessment and risk stratification in inflammatory bowel disease.

## Highlights

• Inflammatory bowel disease was associated with several cardiac differences
• Myocardial thickness-related measures were higher in inflammatory bowel disease
• Systolic and diastolic cardiac indices differed between patients and controls
• Exploratory analyses showed variable subgroup patterns

## Graphical abstract

**Figure:**
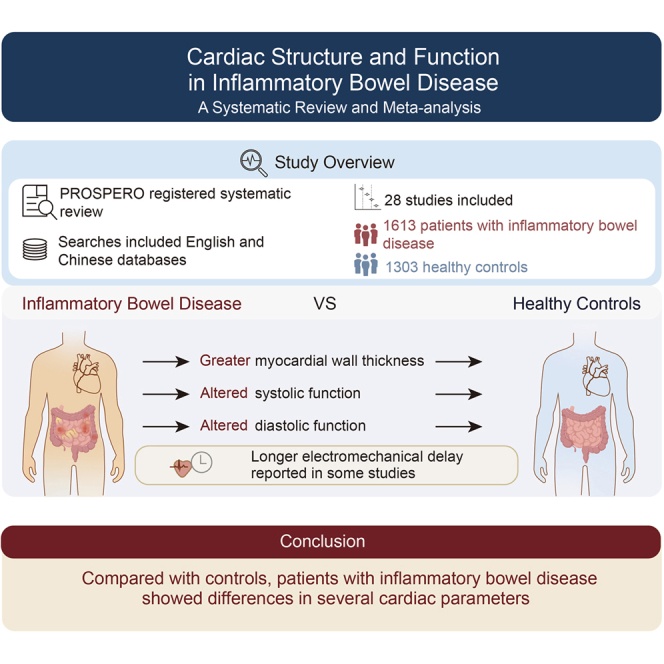

## Introduction

Inflammatory bowel disease (IBD) represents a spectrum of chronic inflammatory disorders mainly affecting the gastrointestinal tract, characterized by alternating episodes of remission and exacerbation. IBD is primarily classified into two distinct clinical entities: ulcerative colitis (UC) and Crohn’s disease (CD). While primarily gastrointestinal, IBD is increasingly recognized as a systemic condition with significant extraintestinal manifestations, affecting various organs, including the cardiovascular (CV) system.

Emerging evidence points to an elevated risk of CV events and poor CV outcomes in individuals with IBD.^1^^,^^2^^,^^3^^,^^4^^,^^5^^,^^6^^,^^7^^,^^8^ However, the exact pathophysiological links between IBD and CV manifestations remain poorly understood.

Recent advancements in echocardiography and magnetic resonance imaging (MRI) have facilitated the detection of subclinical cardiac abnormalities in patients with IBD.^9^^,^^10^ These methods provide quantitative, reproducible insights into ventricular function and structural parameters, enabling a more nuanced assessment of cardiac health. Numerous studies, including a prior systematic review and meta-analysis, have reported differences in cardiac structure and function among individuals with IBD^9^^,^^10^^,^^11^^,^^12^^,^^13^^,^^14^^,^^15^^,^^16^^,^^17^^,^^18^^,^^19^^,^^20^^,^^21^^,^^22^^,^^23^^,^^24^^,^^25^^,^^26^^,^^27^^,^^28^^,^^29^^,^^30^^,^^31^^,^^32^^,^^33^^,^^34^^,^^35^^,^^36^^,^^37^; however, findings across studies remain inconsistent, and the clinical relevance of these differences is still debated.

This meta-analysis aimed to (1) quantify differences in predefined cardiac structural and functional parameters between individuals with IBD and healthy controls, and (2) explore whether these associations vary by key clinical subgroups, including age of onset (pediatric-onset vs. adult-onset), IBD subtype (CD vs. UC vs. mixed IBD), and disease activity (active vs. remission). Electrophysiological timing indices, when available, were evaluated as secondary/exploratory cardiac assessment outcomes. By synthesizing the available evidence, we sought to better characterize the pattern and magnitude of cardiac parameters associated with IBD.

## Results

### Study selection, characteristics, and quality

**Figure 1:**
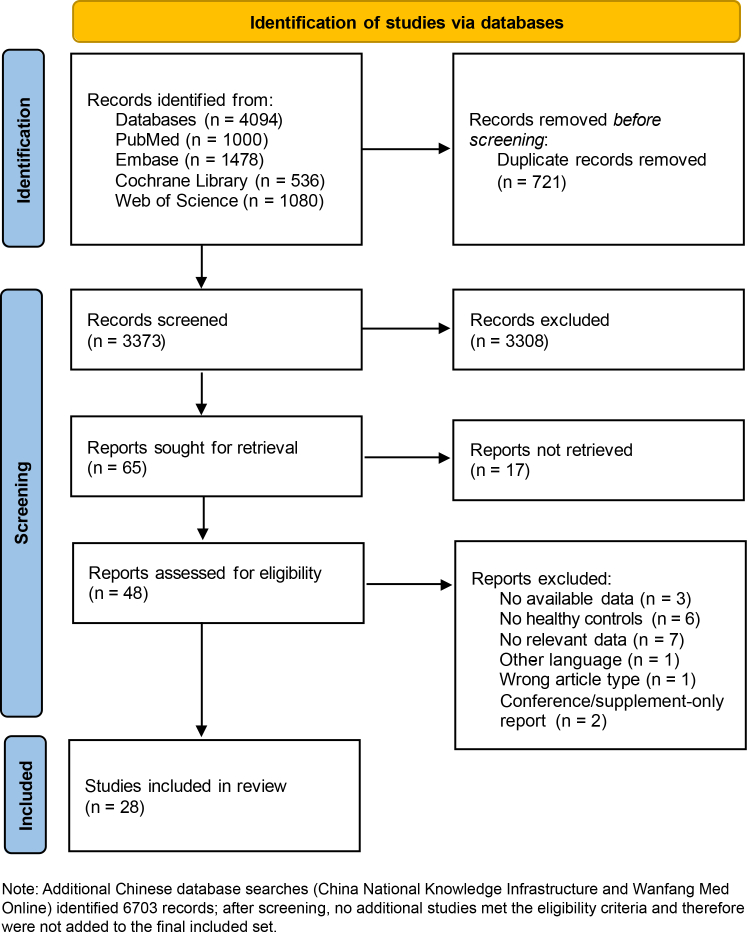
PRISMA flowchart of this study PRISMA, preferred reporting items for systematic reviews and meta-analyses.

Figure 1 displays the workflow of our study. A total of 4094 records were identified through international database searches. After removing duplicates (*n* = 721), 3373 records were screened by title and abstract, and 3308 were excluded. We then sought full texts and/or extractable quantitative outcome data for 65 reports; 17 reports could not be retrieved despite repeated attempts (e.g., searches across alternative platforms and direct author contact). The remaining 48 reports were assessed for eligibility, of which 20 were excluded for the following reasons: wrong article type (*n* = 1), no available quantitative data for extraction (*n* = 3), no healthy controls (*n* = 6), no relevant data for our predefined parameters (*n* = 7), language other than English or Chinese (*n* = 1), and conference abstract/supplement-only report without peer-reviewed full-text publication (*n* = 2). Ultimately, 28 studies met the inclusion criteria and were included in the final meta-analysis.^9^^,^^10^^,^^11^^,^^12^^,^^13^^,^^14^^,^^15^^,^^16^^,^^17^^,^^18^^,^^19^^,^^20^^,^^21^^,^^22^^,^^23^^,^^24^^,^^25^^,^^26^^,^^27^^,^^28^^,^^29^^,^^30^^,^^31^^,^^32^^,^^33^^,^^34^^,^^35^^,^^36^ Additional Chinese database searches identified 6703 records, including 3590 from China National Knowledge Infrastructure and 3113 from Wanfang Med Online; after screening, no additional studies met the eligibility criteria.

Key characteristics of included studies and study populations are summarized in Table 1, with additional methodological details provided in Table S4. All investigations were conducted in countries in Europe, Asia, and Africa. Collectively, our study encompassed 1613 patients with IBD and 1303 healthy controls, including both adults and children. Most cardiac examinations were performed during the remission period (15 studies),^13^^,^^15^^,^^16^^,^^17^^,^^18^^,^^19^^,^^20^^,^^23^^,^^24^^,^^25^^,^^27^^,^^30^^,^^31^^,^^33^^,^^36^ active state (2 studies)^9^^,^^28^ or both (9 studies),^10^^,^^11^^,^^14^^,^^21^^,^^26^^,^^29^^,^^32^^,^^34^^,^^35^ while 2 studies did not report disease activity at the time of cardiac assessment.^12^^,^^22^ A wide range of techniques was employed to assess cardiac structure and function, including conventional echocardiography, speckle tracking echocardiography, and cardiac MRI.

**Table 1:** Characteristics of included studies and study populations

Study ID	Country	Design	Onset age	Type	IBD sample size (n)	IBD age (year)	IBD sex (male/female)	IBD disease activity	Control sample size (n)	Control age (year)	Control sex (male/female)
Bornaun, H. et al.,^16^	Turkey	cross-sectional	pediatric-onset	CD + UC	36	12.37 ± 4.33	17/19	remission state	36	12.11 ± 4.49	17/19
Caliskan, Z. et al.,^21^	Turkey	cross-sectional	adult-onset	CD	30	41 ± 14	16/14	remission state	30	42 ± 8	14/16
Dogan, Y. et al.,^26^	Turkey	cross-sectional	adult-onset	UC + CD	69	39.69 ± 13.21	not reported	severe: 21 mild: 23 moderate: 25	38	41.59 ± 13.12	not reported
Erolu, E. et al.,^30^	Turkey	cross-sectional	pediatric-onset	CD + UC	33	13.3 ± 3.9	17/16	remission state	25	11.6 ± 4	12/13
Kafes, H. et al.,^17^	Turkey	cross-sectional	adult-onset	UC + CD	35	43.97 ± 13.98	18/17	remission state	21	40.14 ± 10.24	15/6
Nemes, A. et al.,^22^	Hungary	Cross-sectional	adult-onset	UC	11	39.0 ± 11.5	7/4	not reported	22	38.8 ± 10.9	11/11
Ozdil, K. et al.,^36^	Turkey	cross-sectional	adult-onset	CD + UC	110	41.1 ± 12.6	49/61	remission state	105	40.8 ± 5.7	59/56
Polat, E. et al.,^20^	Turkey	cross-sectional	pediatric-onset	CD + UC	34	10.9 ± 3.9	18/16	remission state	21	11.6 ± 4.02	10/11
Vizzardi, E. et al.,^13^	Italy	case-control	adult-onset	CD + UC	70	46 ± 16	34/36	remission state	24	43 ± 14	17/7
Sari, C. et al.,^34^	Turkey	prospective	adult-onset	UC + CD	72	40.4 ± 13.1	37/35	active state: 26 remission state: 46	93	37.0 ± 9.2	44/49
Nar, G. et al.,^35^	Turkey	cross-sectional	adult-onset	UC	45	46.8 ± 13.1	32/13	active state: 14 remission state: 31	46	49.5 ± 10.3	29/17
Kivrak, T. et al.,^12^	Turkey	cross-sectional	adult-onset	CD	50	41.0 ± 13.9	24/26	not reported	50	40.1 ± 7.3	24/26
Kahyaoglu, M. et al.,^31^	Turkey	prospective	adult-onset	UC	51	40.2 ± 11.2	29/22	remission state	52	36.2 ± 9.8	34/18
Amrousy, D. et al.,^15^	Egypt	prospective cross-sectional	pediatric-onset	CD + UC	100	16.1 ± 1.9	52/48	remission state	100	15.6 ± 1.8	51/49
Efe, T. H. et al.,^32^	Turkey	cross-sectional	adult-onset	CD + UC	52	active state: 41.4 ± 10.3 remission state: 44.0 ± 10.8	active state: 13/12 remission state: 14/15	active state: 25 remission state: 27	24	37.3 ± 11	13/11
Cincin, A. et al.,^27^	Turkey	cross-sectional	adult-onset	UC	45	36.65 ± 13.81	27/18	remission state	90	39.85 ± 10.68	52/38
Can, G. et al.,^19^	Turkey	cross-sectional, prospective, single-center	adult-onset	CD + UC	79	37.7 ± 11.0	46/33	remission state	70	36.6 ± 8.2	38/32
Caliskan, Z. et al.,^21^	Turkey	cross-sectional	adult-onset	CD + UC	72	39.7 ± 12.1	0.40 ± 0.49	remission state	36	37.2 ± 5.1	0.40 ± 0.49
Aslan, A. N. et al.,^33^	Turkey	cross-sectional	adult-onset	CD + UC	72	41.6 ± 11.7	46/26	remission state	50	39.9 ± 7.9	29/21
Abdelmassih, A. et al.,^11^	Egypt	cross-sectional	pediatric-onset	CD + UC + IBD-U	146	8.76 ± 3.24	94/52	remission state: 84 active state: 62	50	not reported	not reported
Hensel, K. O. et al.,^14^	Germany	cross-sectional	pediatric-onset	CD + UC	50	active state: 14.58 ± 2.51 remission state: 14.3 ± 2.31	active state: 9/9 remission state: 17/15	active state: 18 remission state: 32	60	14.01 ± 2.52	23/37
Hasbey, I. et al.,^10^	Turkey	cross-sectional	adult-onset	CD	20	40.5 ± 7.7	10/10	active state + remission state	20	35.8 ± 13.3	9/11
Fenski, M. et al.,^29^	Germany	prospective	adult-onset	CD + UC	44	39.50 (31.50–58.00)	20/24	CD: active (17), remission (13) UC: active (8), remission (4) indeterminate colitis: active (1), remission (1)	44	38.50 (30.50–53.50)	20/24
Akiya, A. et al.,^18^	Japan	prospective	pediatric-onset	CD + UC	67	UC: 17.7 (15.7–19.5) CD: 18.5 (14.8–21.3)	UC: 26/21 CD: 13/7	remission state	75	16.3 (14.5–20.7)	36/39
Kakuta, K. et al.,^28^	Japan	prospective observational	adult-onset	CD + UC	37	44 ± 15	20/17	active state	30	46 ± 12	20/10
Caliskan, Z. et al.,^25^	Turkey	prospective	adult-onset	CD + UC	101	41 ± 12	45/56	remission state	32	40 ± 4	19/13
Caliskan, Z. et al.,^21^	Turkey	cross-sectional	adult-onset	CD + UC	62	39 ± 11	28/34	active state	39	37 ± 5	26/13
Behairy, A. et al.,^9^	Egypt	cross-sectional	pediatric-onset	UC	20	9.4 ± 3.2	10/10	active state	20	7.5 ± 2.2	6/14

IBD, inflammatory bowel disease; CD, Crohn’s disease; UC, ulcerative colitis; IBD-U, inflammatory bowel disease unclassified.

Table S5 presents the quality assessment results based on the Newcastle-Ottawa Scale (NOS). Overall, the included studies were of moderate to high quality according to the NOS (scores 6–8).

### Cardiac structure parameters

**Figure 2:**
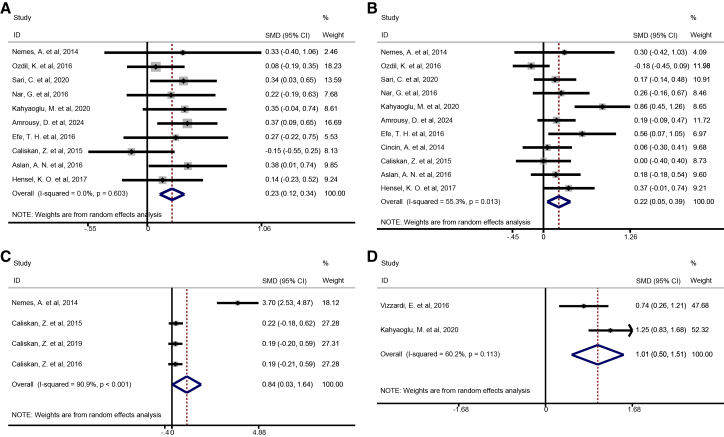
Forest plots compare cardiac structure parameters between patients with IBD and healthy controls (A) Posterior wall. (B) Interventricular septum. (C) Left ventricular mass index. (D) Left atrial volume. IBD, inflammatory bowel disease; SMD, standardized mean difference; CI, confidence interval. The *p*-values shown next to *I^2^* correspond to Cochran’s Q test for heterogeneity.

A total of 2250 individuals across 21 studies that each reported at least one predefined cardiac structural parameter were included in the structural analyses.^9^^,^^10^^,^^11^^,^^14^^,^^15^^,^^16^^,^^17^^,^^21^^,^^23^^,^^24^^,^^25^^,^^27^^,^^32^^,^^33^ Compared with healthy controls, patients with IBD had higher posterior wall (PW) thickness (10 studies; standardized mean difference (SMD) = 0.23, 95% confidence interval (CI): 0.12 to 0.34, *p* < 0.001, *I*^*2*^ = 0.0%) and interventricular septum (IVS) thickness (11 studies; SMD = 0.22, 95% CI: 0.05 to 0.39, *p* = 0.010, *I*^*2*^ = 55.3%) (Figures 2A and 2B). Left ventricular mass index (LVMi) (4 studies; SMD = 0.84, 95% CI: 0.03 to 1.64, *p* = 0.043, *I*^*2*^ = 90.9%) and left atrial (LA) volume (2 studies; SMD = 1.01, 95% CI: 0.50 to 1.51, *p* < 0.001, *I*^*2*^ = 60.2%) were also higher in patients with IBD (Figures 2C and 2D).

Other predefined structural parameters did not differ significantly between patients with IBD and healthy controls, including left ventricular (LV) end-diastolic diameter (SMD = 0.13, 95% CI: −0.09 to 0.35, *p* = 0.235, *I*^*2*^ = 74.7%), LV end-systolic diameter (LVESD) (SMD = 0.15, 95% CI: −0.07 to 0.37, *p* = 0.183, *I*^*2*^ = 72.4%) (both assessed in more than 10 studies), LA diameter (SMD = 0.08, 95% CI: −0.07 to 0.24, *p* = 0.294, *I*^*2*^ = 27.4%), LV end-diastolic volume (SMD = 0.05, 95% CI: −0.20 to 0.30, *p* = 0.687, *I*^*2*^ = 48.3%), LV end-systolic volume (SMD = 0.22, 95% CI: −0.02 to 0.45, *p* = 0.075, *I*^*2*^ = 0.0%) and LV mass (SMD = 0.01, 95% CI: −0.31 to 0.34, *p* = 0.932, *I*^*2*^ = 0.0%) (Figures S1A, S1B, S1C, S1D, S1E, and S1F).

### Cardiac function parameters

All included studies reported one or more predefined cardiac function parameters, encompassing LV systolic and diastolic function, right ventricular function, and overall myocardial performance.

**Figure 3:**
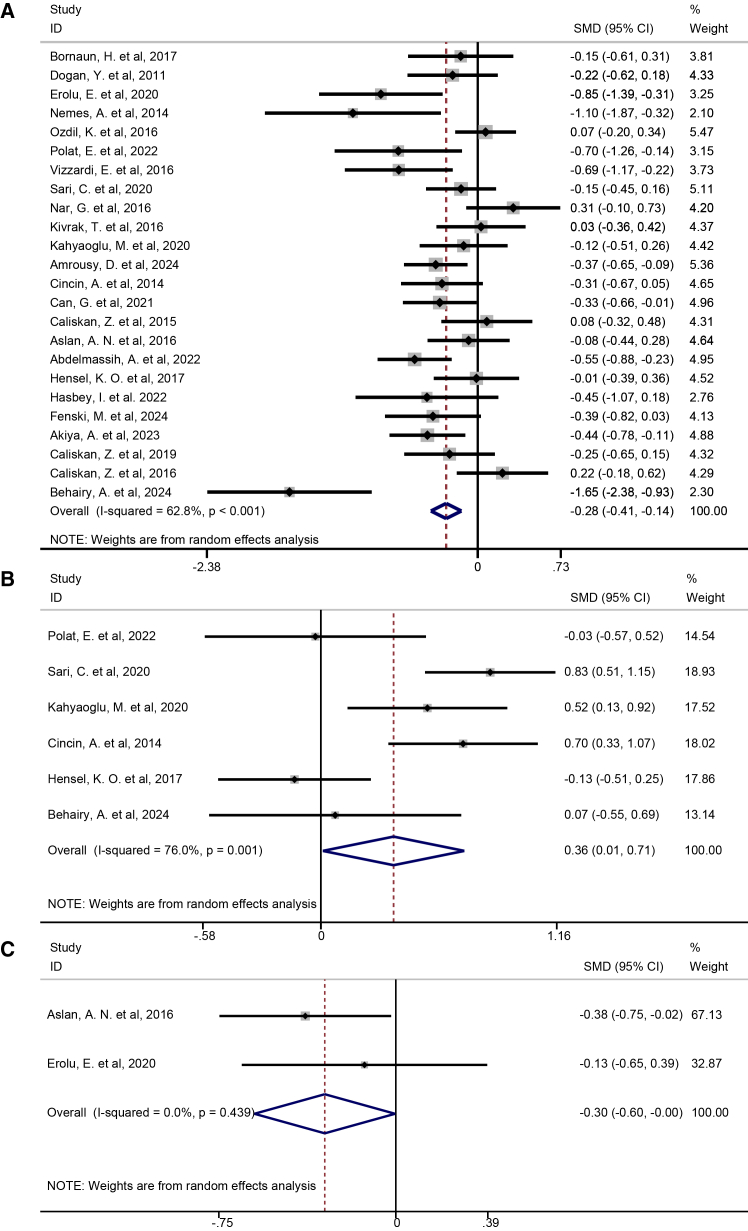
Forest plots compare cardiac systolic parameters between patients with IBD and healthy controls (A) Ejection fraction. (B) Global longitudinal strain. (C) Isovolumetric contraction time. IBD, inflammatory bowel disease; SMD, standardized mean difference; CI, confidence interval. The *p*-values shown next to *I^2^* correspond to Cochran’s Q test for heterogeneity.

As for LV systolic function, ejection fraction (EF) was reported in 24 studies and was significantly lower in patients with IBD than in healthy controls (SMD = −0.28, 95% CI: −0.41 to −0.14, *p* < 0.001, *I*^*2*^ = 62.8%) (Figure 3A). Fractional shortening (FS) did not differ significantly between patients with IBD and healthy controls (SMD = −0.21, 95% CI: −0.60 to 0.19, *p* = 0.303, *I*^*2*^ = 67.5%) (Figure S2A). Global longitudinal strain (GLS) was less negative in patients with IBD (SMD = 0.36, 95% CI: 0.01 to 0.71, *p* = 0.044, *I*^*2*^ = 76.0%) (Figure 3B), whereas global circumferential strain did not exhibit any notable difference (SMD = 0.22, 95% CI: −0.08 to 0.53, *p* = 0.144, *I*^*2*^ = 56.4%) (Figure S2B). Isovolumetric contraction time was shorter in patients with IBD (SMD = −0.30, 95% CI: −0.60 to −0.00, *p* = 0.048, *I*^*2*^ = 0.0%) (Figure 3C).

**Figure 4:**
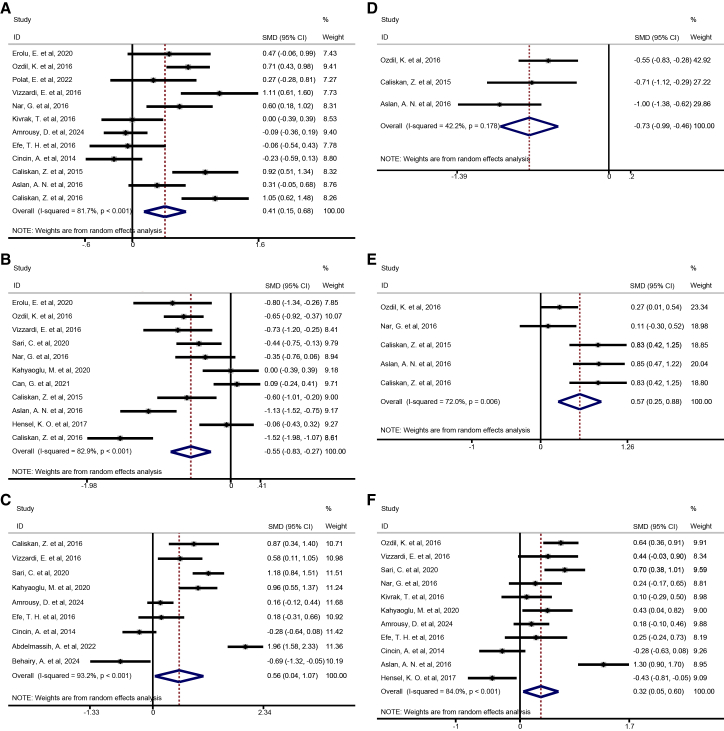
Forest plots compare cardiac diastolic parameters between patients with IBD and healthy controls (A) A wave. (B) E/A ratio. (C) E/E′ ratio. (D) E'/A′ ratio. (E) Isovolumetric relaxation time. (F) Mitral deceleration time. IBD, inflammatory bowel disease; SMD, standardized mean difference; CI, confidence interval. The *p*-values shown next to *I^2^* correspond to Cochran’s Q test for heterogeneity.

Among the LV diastolic parameters evaluated, the A wave was higher in patients with IBD (SMD = 0.41, 95% CI: 0.15 to 0.68, *p* = 0.002, *I*^*2*^ = 81.7%) (Figure 4A). Key ratios such as E/A ratio (SMD = −0.55, 95% CI: −0.83 to −0.27, *p* < 0.001, *I*^*2*^ = 82.9%), E/E′ ratio (SMD = 0.56, 95% CI: 0.04 to 1.07, *p* = 0.034, *I*^*2*^ = 93.2%), and E'/A′ ratio (SMD = −0.73, 95% CI: −0.99 to −0.46, *p* < 0.001, *I*^*2*^ = 42.2%) also differed significantly between patients with IBD and healthy controls (Figures 4B–4D). Moreover, both isovolumetric relaxation time (IVRT) (SMD = 0.57, 95% CI: 0.25 to 0.88, *p* < 0.001, *I*^*2*^ = 72.0%) and mitral deceleration time (DT) (SMD = 0.32, 95% CI: 0.05 to 0.60, *p* = 0.021, *I*^*2*^ = 84.0%) were elevated in patients with IBD (Figures 4E and 4F). By contrast, the E wave (SMD = −0.26, 95% CI: −0.53 to 0.00, *p* = 0.054, *I*^*2*^ = 83.2%), A′ wave (SMD = 0.22, 95% CI: −0.18 to 0.62, *p* = 0.273, *I*^*2*^ = 85.2%), and E′ wave (SMD = −0.49, 95% CI: −1.11 to 0.12, *p* = 0.117, *I*^*2*^ = 95.3%) did not exhibit statistically significant differences between patients with IBD and healthy controls (Figures S3A, S3B, and S3C).

**Figure 5:**
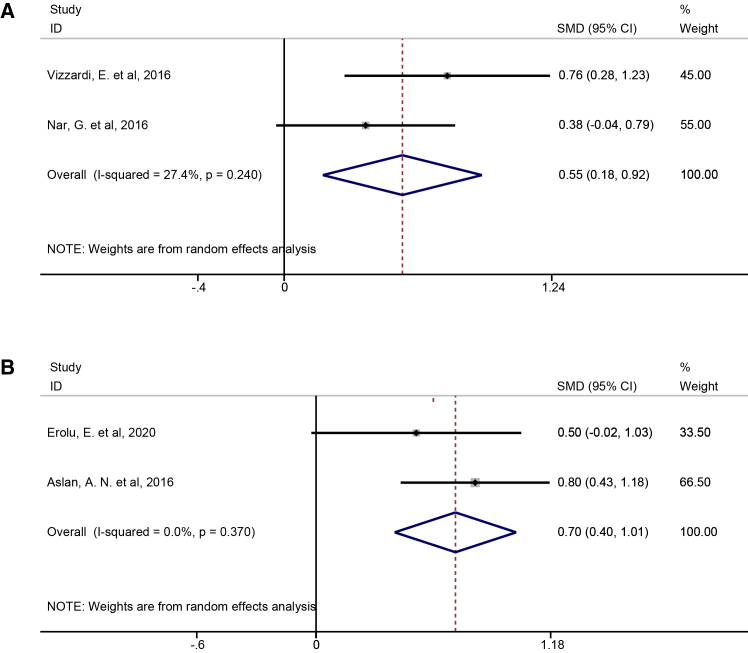
Forest plots compare other cardiac parameters between patients with IBD and healthy controls (A) Pulmonary artery systolic pressure. (B) Myocardial performance index. IBD, inflammatory bowel disease; SMD, standardized mean difference; CI, confidence interval. The *p*-values shown next to *I^2^* correspond to Cochran’s Q test for heterogeneity.

Two studies assessed right ventricular EF and found no significant difference between patients with IBD and healthy controls (SMD = −0.41, 95% CI: −1.70 to 0.87, *p* = 0.528, *I*^*2*^ = 90.6%) (Figure S2C). Pulmonary artery systolic pressure, reported in two studies, was higher in patients with IBD (SMD = 0.55, 95% CI: 0.18 to 0.92, *p* = 0.004, *I*^*2*^ = 27.4%) (Figure 5A).

Overall myocardial performance, as characterized by myocardial performance index (MPI), was evaluated in two studies and was higher in patients with IBD (SMD = 0.70, 95% CI: 0.40 to 1.01, *p* < 0.001, *I*^*2*^ = 0.0%) (Figure 5B).

### Secondary/exploratory outcomes

**Figure 6:**
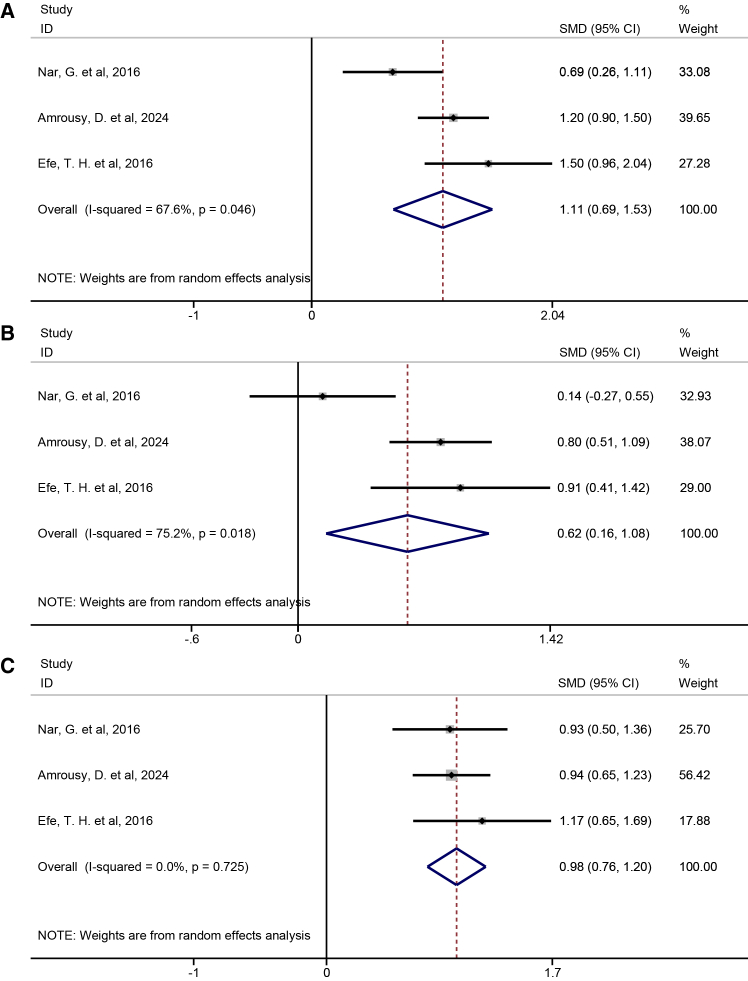
Forest plots compare cardiac electrophysiological parameters between patients with IBD and healthy controls (A) Inter-atrial electromechanical delay. (B) Intra-left electromechanical delay. (C) Intra-right electromechanical delay. IBD, inflammatory bowel disease; SMD, standardized mean difference; CI, confidence interval. The *p*-values shown next to *I^2^* correspond to Cochran’s Q test for heterogeneity.

As secondary/exploratory outcomes, electrophysiological timing indices were reported in three studies.^15^^,^^32^^,^^35^ Notably, inter-atrial electromechanical delay (EMD) (SMD = 1.11, 95% CI: 0.69 to 1.53, *p* < 0.001, *I*^*2*^ = 67.6%), intra-left EMD (SMD = 0.62, 95% CI: 0.16 to 1.08, *p* = 0.008, *I*^*2*^ = 75.2%), and intra-right EMD (SMD = 0.98, 95% CI: 0.76 to 1.20, *p* < 0.001, *I*^*2*^ = 0.0%) were all significantly longer in patients with IBD (Figures 6A–6C).

### Subgroup analysis

Subgroup analyses were performed to explore potential sources of substantial heterogeneity (*I*^*2*^ > 50%) and to examine subgroup patterns by age of onset, IBD subtype, and disease activity; these analyses were exploratory. Full subgroup-specific pooled estimates are provided in Figures S4, S5, S6, S7, S8, S9, S10, S11, S12, S13, S14, S15, S16, and S17, and key estimates are summarized later in discussion to support the main patterns described in the text.

Across pediatric-onset and adult-onset subgroups, most parameters showed trends broadly consistent with the overall analysis. In adult-onset studies, pooled estimates suggested differences for GLS (SMD = 0.71, 95% CI: 0.50–0.91), A wave (SMD = 0.48, 95% CI: 0.17–0.80), E/A ratio (SMD = −0.58, 95% CI: −0.89 to −0.27), E/E′ ratio (SMD = 0.58, 95% CI: 0.08–1.07), and DT (SMD = 0.43, 95% CI: 0.14–0.72) (Figures S10–S14). In pediatric-onset studies, pooled differences were observed for some parameters that were not statistically significant in the overall analysis, such as LVESD (SMD = 2.10, 95% CI: 1.32–2.88), FS (SMD = −0.36, 95% CI: −0.70 to −0.01), and E wave (SMD = −0.27, 95% CI: −0.49 to −0.04); however, these findings were based on a limited number of studies and should be interpreted cautiously (Figures S7, S9 and S15).

Patterns also varied across IBD subtypes. For IVS and GLS, which differed between patients with IBD and healthy controls in the overall analysis, pooled estimates were most evident in the UC subgroup (IVS: SMD = 0.29, 95% CI: 0.03–0.56; GLS: SMD = 0.38, 95% CI: 0.02–0.74) (Figures S4 and S10). In studies with mixed IBD populations, pooled differences were observed for several LV diastolic parameters, including A wave (SMD = 0.60, 95% CI: 0.28–0.93), E/A ratio (SMD = −0.75, 95% CI: −1.08 to −0.41), E/E′ ratio (SMD = 0.99, 95% CI: 0.25–1.72), and DT (SMD = 0.49, 95% CI: 0.04–0.93) (Figures S11–S14). For certain outcomes, such as LVESD and E wave, pooled differences were observed within the UC subgroup but not in other subtype strata (Figures S7 and S15). By contrast, LVMi—significant in the overall analysis—was not statistically significant within individual subgroups after stratification (Figure S5).

Regarding disease activity, pooled estimates suggested differences in several parameters in studies assessing patients in remission, including EF (SMD = −0.27, 95% CI: −0.39 to −0.14), GLS (SMD = 0.39, 95% CI: 0.05–0.74), A wave (SMD = 0.39, 95% CI: 0.08–0.70), E/A ratio (SMD = −0.47, 95% CI: −0.79 to −0.16), and E/E′ ratio (SMD = 0.40, 95% CI: 0.00–0.80) (Figures S8 and S10–S13). By contrast, pooled estimates in studies assessing active disease did not reach statistical significance, although these strata included few studies and should be interpreted cautiously.

In addition, exploratory univariable meta-regression for EF showed that age of onset (pediatric-onset vs. adult-onset) was associated with EF effect estimates (*β* = −0.33, 95% CI: −0.64 to −0.02, *p* = 0.037, *k* = 24), whereas IBD subtype (subtype-specific UC/CD vs. mixed IBD cohort) and disease activity were not (all *p* > 0.70) (Table S6).

### Publication bias and sensitivity analysis

The potential for publication bias was evaluated through the visual inspection of funnel plot symmetry and statistical analysis using Egger’s test when ≥10 studies were available for an outcome. Most outcomes showed no clear asymmetry in funnel plots, and Egger’s tests yielded non-significant results (*p* > 0.05). EF was the only outcome with a significant Egger’s test result (*p* = 0.016).

Sensitivity analysis, performed utilizing the leave-one-out (LOO) method, further evaluated the robustness of the findings. The results demonstrated that the overall effect estimates for most parameters were stable and not notably affected by the exclusion of individual studies. For EF, the pooled estimate remained directionally consistent and statistically significant after omitting individual studies. However, GLS and DT exhibited study-dependent variability, with pooled estimates becoming less precise and no longer statistically significant when excluding the study by *Sari et al*.^34^ Additionally, parameters assessed in a limited number of studies (e.g., LVMi, MPI, and intra-left EMD) were highly sensitive to the omission of individual studies.

## Discussion

Although IBD is a recognized risk factor for CV disease and is linked to an increased risk of CV events,^38^^,^^39^^,^^40^ the presence of structural and functional cardiac involvement in IBD remains controversial, and the underlying mechanisms remain incompletely understood.

Compared with the prior systematic review and meta-analysis by *Soares et al.*, which searched PubMed and Scopus up to September 2022 and included 18 studies in the qualitative synthesis and 9 studies in the quantitative meta-analysis,^37^ our review used a broader search strategy and a more imaging-focused quantitative framework. Based on the studies listed in their outcome-level quantitative analyses, all nine studies contributing to the prior meta-analysis were included in our quantitative synthesis,^12^^,^^19^^,^^24^^,^^27^^,^^31^^,^^32^^,^^33^^,^^34^^,^^35^ and 10 of the 18 studies in their qualitative review were also included in our review.^10^^,^^12^^,^^19^^,^^24^^,^^27^^,^^31^^,^^32^^,^^33^^,^^34^^,^^35^ The non-overlapping studies from the prior qualitative review mainly addressed heart failure hospitalization, broader CV events, treatment-related CV risk, vascular outcomes, or did not provide extractable IBD-versus-healthy-control data for our predefined cardiac structural and functional parameters. Conversely, our review included 18 additional eligible studies and quantified a broader range of cardiac structural and functional domains.^9^^,^^11^^,^^13^^,^^14^^,^^15^^,^^16^^,^^17^^,^^18^^,^^20^^,^^21^^,^^22^^,^^23^^,^^25^^,^^26^^,^^28^^,^^29^^,^^30^^,^^36^

Within this expanded evidence base, our findings largely replicated the main echocardiographic patterns reported by *Soares et al.*, including lower E/A ratio, higher E/E′ ratio, impaired GLS, longer EMD indices, and no significant difference in LA diameter.^37^ Our analysis further extended these findings by quantifying additional structural, systolic, and diastolic domains. Notably, structural differences included higher PW thickness, IVS thickness, LVMi, and LA volume, while functional differences included lower EF and higher MPI. Taken together, these findings suggest that IBD is associated with modest differences across structural and functional cardiac domains, and advanced measurement techniques, such as speckle tracking echocardiography, may be useful for detecting subclinical abnormalities.^9^ However, individual parameter-level findings should be interpreted cautiously because several outcomes were related measures from the same cardiac assessments, and no formal multiplicity adjustment was applied. Electrophysiological timing indices were retained as secondary/exploratory cardiac assessment outcomes because prior studies in IBD have suggested that atrial EMD may reflect subclinical atrial conduction-mechanical coupling abnormalities and possible susceptibility to atrial arrhythmia in a small subset of patients.^15^^,^^19^^,^^32^

Regarding variability in outcomes, some cardiac parameters showed statistically significant differences, while others did not. This pattern may reflect differences in the sensitivity of individual parameters to detect subclinical differences, as well as variation in measurement protocols and residual confounding (e.g., medication use and comorbidities). Exploratory subgroup analyses showed variable patterns across age of onset, IBD subtype, and disease activity, but these descriptive patterns should not be interpreted as definitive evidence that these factors explain between-study heterogeneity. Formal meta-regression was feasible only for EF; in that analysis, age of onset was associated with EF effect estimates, whereas IBD subtype and disease activity were not. Collectively, these results suggest that patient- and disease-related characteristics may be relevant to cardiac parameters in IBD, but their role in explaining heterogeneity requires confirmation in larger, more consistently reported datasets. Future studies may benefit from standardized cardiac assessment protocols and more granular stratification by disease type, activity, and key covariates to better clarify how these factors relate to CV parameters in IBD.

The pathophysiology underlying the observed associations between IBD and cardiac abnormalities is likely multifactorial.^41^ Systemic inflammation has been proposed as a key contributor.^42^ IBD is associated with elevated pro-inflammatory cytokines (e.g., TNF-α, IL-6, and IL-1β),^43^ which have been linked to myocardial remodeling, endothelial dysfunction, and increased vascular stiffness.^44^ Gut microbiota dysbiosis, a hallmark of IBD, may further influence CV health by increasing intestinal permeability and facilitating the translocation of bacterial endotoxins and antigens into circulation, potentially amplifying systemic inflammation. Furthermore, dysregulated microbiota-derived metabolites (e.g., reduced short-chain fatty acids and elevated trimethylamine-N-oxide) may exacerbate inflammation and promote adverse cardiac remodeling.^45^ Collectively, these mechanisms suggest that gut dysbiosis may be relevant to CV involvement in IBD. IBD immunosuppressive therapies (particularly corticosteroids) and comorbid conditions (e.g., metabolic syndrome, anemia, and malnutrition) may also contribute to CV risk profiles and could confound or modify the observed associations.^46^^,^^47^^,^^48^ Further mechanistic and longitudinal studies are needed to delineate these pathways and to clarify their clinical implications.

In conclusion, this systematic review and meta-analysis suggests that IBD is associated with modest differences across structural and functional cardiac domains, including myocardial thickness-related measures and indices of systolic and diastolic function; limited secondary/exploratory data also suggested longer electromechanical timing indices where reported. Advanced imaging techniques (e.g., speckle tracking echocardiography) may be useful for detecting subclinical cardiac abnormalities in this context. However, interpretation is limited by heterogeneity, residual confounding, and the predominance of cross-sectional observational evidence. Proposed mechanisms—including systemic inflammation, gut dysbiosis, and treatment-related effects—may contribute to these findings but require further confirmation. Longitudinal studies with standardized cardiac assessment and comprehensive reporting are warranted to clarify the long-term CV implications of IBD and to inform risk stratification and management strategies.

### Limitations of the study

While this investigation provides significant contributions to the field, the interpretation of the findings warrants caution. The primary limitation stems from the predominance of cross-sectional study designs among the included studies, which precludes the determination of causal associations. Additionally, the observational nature of the evidence introduces inherent biases and variability. Although the included studies were generally of moderate-to-high quality according to the NOS, domain-specific risks of bias typical of observational research remain, particularly related to selection (e.g., representativeness of cases and the selection of controls) and residual confounding. In addition, because our quantitative synthesis was based on extracted means and standard deviations for patients with IBD and healthy controls rather than adjusted study-specific effect estimates, any statistical adjustment performed in the primary studies was not directly carried forward into the pooled analyses. Even when some studies reported age/sex-matched or otherwise selected healthy controls, residual confounding cannot be excluded because matching and control selection procedures were not uniform across studies, and adjustment for limited covariates in the primary studies may not have fully addressed these differences. In addition, sex- or ethnicity-specific associations could not be evaluated because stratified cardiac outcome data were not consistently reported in the primary studies. Another important limitation is that this review synthesized multiple related cardiac parameters within predefined domains rather than prioritizing one or two primary outcomes, and no formal multiplicity adjustment was applied. Because several outcomes were physiologically related and often derived from the same cardiac assessments, individual parameter-level findings should be interpreted as exploratory and considered mainly as signals within broader cardiac domains rather than as multiple independent associations. Importantly, non-significant pooled results—especially for outcomes reported by few studies—should not be interpreted as evidence of no association. Similarly, subgroup-specific findings may be unstable when based on few studies and imprecise estimates, and should not be interpreted as definitive effect modification. Variations in study methodologies, such as differences in patient populations, diagnostic criteria, and measurement techniques (e.g., echocardiographic assessments), further contribute to heterogeneity. Although exploratory meta-regression (performed only for EF) suggested that age of onset was associated with EF effect estimates, these analyses were limited by incomplete reporting of study-level covariates and sparse categories for some factors, and substantial residual heterogeneity remained. Accordingly, heterogeneity is likely driven by additional unmeasured clinical and methodological differences across studies. Technique-based heterogeneity exploration was also limited by inconsistent reporting of imaging methods and sparse cardiac MRI data (two studies), precluding formal modality-based subgroup testing. The lack of standardized protocols for assessing cardiac parameters may also affect the consistency and reliability of the results. Moreover, differences in treatment regimens, particularly immunosuppressive therapies, corticosteroid use, and comorbidities (e.g., metabolic syndrome or anemia), may have confounded the observed associations, complicating interpretation. In addition, missing data and selective reporting of outcomes cannot be excluded, as not all studies reported the same set of cardiac parameters with complete summary statistics. The significant Egger’s test result observed for EF may reflect small-study effects or selective reporting and should be interpreted cautiously given residual heterogeneity and methodological differences across included studies; however, the pooled EF estimate remained directionally consistent and statistically significant in LOO sensitivity analysis. Moreover, transformed (e.g., ln/log10) summary statistics were not reported or were too sparse for our predefined imaging outcomes, limiting our ability to synthesize results on a transformed scale. Importantly, despite repeated attempts to retrieve full texts and obtain extractable quantitative data, some potentially relevant reports and/or usable outcome data were unavailable for pooling. This incomplete availability of evidence may introduce availability (selection) bias and may have affected the precision and representativeness of the pooled estimates. In addition, the body of evidence on detailed cardiac imaging parameters in IBD remains relatively limited and is largely derived from single-center observational studies, many of which are published in specialty journals. While IBD-related CV risk is increasingly recognized, high-profile multicenter studies have more often focused on clinical CV events rather than comprehensive echocardiographic/cardiac MRI phenotyping. This likely contributes to the modest number of eligible comparative studies and the characteristics of the cited literature.

## Resource availability

### Lead contact

Further information and requests for resources should be directed to and will be fulfilled by the lead contact, Ying Xiao (xiaoying1113@csu.edu.cn).

### Materials availability

This study did not generate new unique materials.

### Data and code availability

• All data reported in this paper are included in the article and supplemental information. Outcome-level extracted data used for the meta-analyses are provided in Table S3. This study did not generate new standardized datasets.
• This paper does not report original code.
• Any additional information required to reanalyze the data reported in this paper is available from the lead contact upon request.

## Acknowledgments

This work was supported by the 10.13039/501100001809National Natural Science Foundation of China (nos. 82230019, 82341225, and 82300640) and the 10.13039/501100004735Natural Science Foundation of Hunan Province (no. 2024JJ6674).

## Author contributions

Y.Y.: conceptualization, methodology, investigation, visualization, and writing – original draft preparation. X.L.: investigation and writing – original draft preparation. L.Z.: investigation and validation. G.S.: writing – reviewing and editing and validation. R.Y.: writing – reviewing and editing, visualization, validation, and data curation. Y.X.: writing – reviewing and editing and supervision. X.L.: writing – reviewing and editing and supervision.

## Declaration of interests

The authors declare no competing interests.

## Declaration of generative AI and AI-assisted technologies in the writing process

The authors declare that no generative AI or AI-assisted technologies were used in the preparation of this work.

## STAR★Methods

### Key resources TABLE

**Table:** 

REAGENT or RESOURCE	SOURCE	IDENTIFIER
**Deposited data**		

Studies for meta-analysis	Embase	https://www.embase.com/
Studies for meta-analysis	PubMed	https://pubmed.ncbi.nlm.nih.gov/
Studies for meta-analysis	Cochrane Library	https://www.cochranelibrary.com/advanced-search
Studies for meta-analysis	Web of Science Core Database	https://www.webofscience.com/wos/author/search
Studies for meta-analysis	China National Knowledge Infrastructure	https://www.cnki.net
Studies for meta-analysis	Wanfang Med Online	https://med.wanfangdata.com.cn
International prospective register of systematic reviews	PROSPERO	https://www.crd.york.ac.uk/PROSPERO/

**Software and algorithms**		

Stata software 12.0	StataCorp LLC	https://www.stata.com
EndNote *X*9	Clarivate Analytics	https://endnote.com

### Experimental model and study participant details

This systematic review and meta-analysis synthesized published aggregate data and did not involve new recruitment of human participants, animal experiments, or cell-line studies; it was not a clinical trial, and institutional ethics approval was therefore not required.

### Method details

#### Study design and search strategy

This study adhered to the Preferred Reporting Items for Systematic Reviews and Meta-Analyses guidelines (Table S1) and was conducted based on a predefined protocol registered in PROSPERO (PROSPERO: CRD42024577085, registered on 19 August 2024). Two independent reviewers (YY and LX) systematically reviewed PubMed, Embase, Cochrane Library and Web of Science for publications from the inception of each database up to 9 May 2026.

The search terms encompassed keywords related to IBD and cardiac structure and function, alongside relevant echocardiographic indicators. A detailed search strategy can be found in Table S2. We also searched China National Knowledge Infrastructure and Wanfang Med Online using equivalent Chinese search terms up to 9 May 2026 (Data S1). Reference lists of included studies and relevant reviews were additionally screened to identify any potentially eligible articles missed by database searches.

#### Eligibility criteria

Studies were eligible if they met all of the following criteria: (1) included patients with IBD (UC and/or CD); (2) included healthy controls; and (3) reported at least one predefined cardiac parameter with extractable quantitative data for both IBD patients and healthy controls. The primary study aim did not need to be cardiac-related, provided that relevant parameters were reported with sufficient data for synthesis.

Predefined eligible cardiac parameters were specified a priori and grouped into four domains. (1) Cardiac structure: PW thickness, IVS thickness, LV mass and/or LVMi, LA size (LA diameter and/or LA volume), and LV dimensions/volumes (e.g., LV end-diastolic diameter, LVESD, LV end-diastolic volume and LV end-systolic volume), where available. (2) LV systolic function: EF, FS, speckle-tracking–derived strain indices (e.g., GLS; global circumferential strain when reported), and systolic timing indices (e.g., isovolumetric contraction time). (3) LV diastolic function: transmitral inflow indices (E wave, A wave, E/A ratio), tissue Doppler indices (E′ wave, A′ wave, E/E′ ratio, E'/A′ ratio), and other diastolic measures when reported (e.g., mitral DT and IVRT). (4) Other cardiac assessment outcomes: right ventricular EF, pulmonary artery systolic pressure, and MPI. Secondary/exploratory outcomes (if reported): electrophysiological timing indices (e.g., EMD).

Exclusion criteria were: (1) non-original articles (reviews, editorials, letters), and conference abstracts/supplement-only reports without sufficient peer-reviewed full-text data; (2) publications in languages other than English or Chinese; (3) studies without healthy controls; (4) studies lacking sufficient quantitative data for pooling (e.g., missing dispersion measures such as standard deviation, non-convertible statistics, or unavailable data after author contact); and (5) outcomes with clearly non-interpretable or internally inconsistent summary statistics (e.g., values inconsistent with tables/figures, implausible dispersion measures, or irreconcilable unit/reporting errors). Such cases were independently double-checked and, when feasible, clarified by contacting authors; if unresolved, only the affected outcome was excluded from quantitative synthesis rather than excluding the entire study whenever possible.

#### Study selection and data extraction

The process of selecting studies and extracting data was carried out independently by two reviewers (YY and LX) under the supervision of an expert in the field, following the Preferred Reporting Items for Systematic Reviews and Meta-Analyses protocol. Initially, duplicate records were identified and removed. Subsequently, the remaining records underwent title and abstract assessment to determine their potential eligibility based on established selection criteria. Studies meeting the preliminary eligibility requirements were then comprehensively reviewed to confirm their suitability for final inclusion. Importantly, when multiple records were found to report results from the same underlying study population (e.g., conference/supplement records subsequently published as full-length articles, or duplicate/overlapping publications), we linked these reports to a single study and extracted data only once, prioritizing the most complete peer-reviewed full-text publication to avoid double counting.

Data extraction was performed using a standardized template to ensure consistency and comprehensiveness. Extracted information included study characteristics, clinical factors and cardiac parameters. Extracted outcomes were organized according to the predefined domains of cardiac structure, LV systolic function, LV diastolic function, other cardiac assessment outcomes and secondary/exploratory outcomes, and all eligible outcomes were extracted and analyzed irrespective of statistical significance. When studies reported stratified results (e.g., by IBD subtype or disease activity), we extracted subgroup-specific summary data whenever available; if stratified data were not reported, we contacted corresponding authors to request them, and otherwise analyzed the study using the overall estimates.

Outcome definitions. For diastolic function, transmitral inflow indices were defined as the peak early diastolic transmitral flow velocity (E wave) and peak late diastolic transmitral flow velocity (A wave); tissue Doppler indices were defined as the early (E′) and late (A′) diastolic mitral annular velocities. DT was defined as mitral E-wave deceleration time, and IVRT as the interval between aortic valve closure and mitral valve opening, as reported in each study. For structural parameters, PW thickness and IVS thickness were extracted (end-diastole when reported, or according to standard echocardiographic convention when not explicitly stated). When reporting differed across studies, we extracted the most comparable metric to enable synthesis.

Detailed outcome-level extracted data used for the meta-analyses are provided in Table S3. Reviewer disagreements during study selection or data extraction were adjudicated by an independent third reviewer (ZL), ensuring the inclusion of high-quality studies and enhancing review reliability.

#### Risk of bias and quality assessment

Two independent reviewers (YY and LX) evaluated the quality of all eligible studies by applying the NOS. Studies were assessed across three domains: selection, comparability, and exposure. We used the NOS to facilitate comparability with prior reviews; however, consistent with methodological guidance, we did not rely on a single overall score alone to draw conclusions about risk of bias. Instead, we also considered domain-specific sources of bias relevant to observational studies, including missing data, selective reporting of outcomes, potential confounding (e.g., comorbidities and treatments), exposure classification (IBD subtype/activity), and outcome measurement (e.g., echocardiographic protocol variability and blinding). Any discrepancies were resolved by discussion and adjudication by a third reviewer (ZL).

Publication bias was assessed by visual inspection of funnel plot symmetry and Egger’s test when ≥10 studies were available for an outcome. The robustness of the results was further evaluated using a LOO approach, iteratively omitting one study at a time and re-estimating the pooled effect. To explore potential contributors to heterogeneity, subgroup analyses were conducted by stratifying studies according to age of onset (pediatric-onset vs. adult-onset IBD), IBD subtypes (CD vs. UC vs. mixed IBD), and disease activity (active vs. remission), with pooled estimates calculated within each subgroup; subgroup results were compared descriptively. Because modality reporting was inconsistent and cardiac MRI data were sparse (two studies), formal modality-based subgroup testing was not feasible; therefore, measurement technique was recorded as reported.

### Quantification and statistical analysis

Statistical analyses were performed with STATA software 12.0. Given the anticipated heterogeneity resulting from diverse study designs and population characteristics, a random-effects model was utilized. Cardiac parameters were analyzed by computing SMDs with 95% CIs. Meta-analyses were performed separately for each predefined outcome, using all studies that reported that specific parameter with extractable quantitative data; therefore, an individual study could contribute to some outcomes but not others. Because multiple related outcomes were evaluated across predefined cardiac domains, no formal multiplicity adjustment was applied. Accordingly, parameter-level estimates were interpreted descriptively and in the context of broader cardiac domains rather than as independent associations. For strain measures that are conventionally reported as negative values (e.g., GLS), we retained the original sign as reported; therefore, a positive SMD indicates a less negative (i.e., worse) strain in IBD patients compared with controls. Heterogeneity was assessed using Cochran’s Q test and quantified with *I*^*2*^; the *p*-values shown next to *I*^*2*^ in the forest plots correspond to the Q test. Statistical significance for the overall effect was set at *p* < 0.05. In the forest plots, *p*-values reported as 0.000 by the software are presented as *p* < 0.001. As an additional heterogeneity exploration for EF (the most frequently reported outcome), we performed exploratory univariable meta-regression using the restricted maximum likelihood estimator with Knapp–Hartung adjustment to test whether prespecified study-level factors (age of onset, IBD subtype, and disease activity, where available) were associated with EF effect estimates.

## Supplemental information

Document S1. Figures S1–S17, Tables S1, and Data S1

Table S2. Literature search strategy of this study

Table S3. Outcome-level extracted data used for the meta-analyses

Table S4. Study-level methodological details of included studies

Table S5. Quality scores according to the Newcastle-Ottawa Scale

Table S6. Exploratory univariable meta-regression for EF (SMD) using the restricted maximum likelihood estimator with Knapp–Hartung adjustment EF, ejection fraction. SMD, standardized mean difference.
