# Supplementary material for: Cardiac structure and function in patients with inflammatory bowel disease
Source: iScience. 2026 Jun 27;29(7):116524. doi: 10.1016/j.isci.2026.116524 (PMC13378013; doi:10.1016/j.isci.2026.116524)
Supplement: Document S1. Figures S1–S17, Tables S1, and Data S1 [file mmc1.pdf]

iScience, Volume *29*

Supplemental information

Cardiac structure and function in patients with inflammatory bowel disease

Yang Yang, Xingyang Luo, Linxiang Zhou, Guanzhong Shi, Ruozhu Yang, Ying Xiao, and Xiaowei Liu

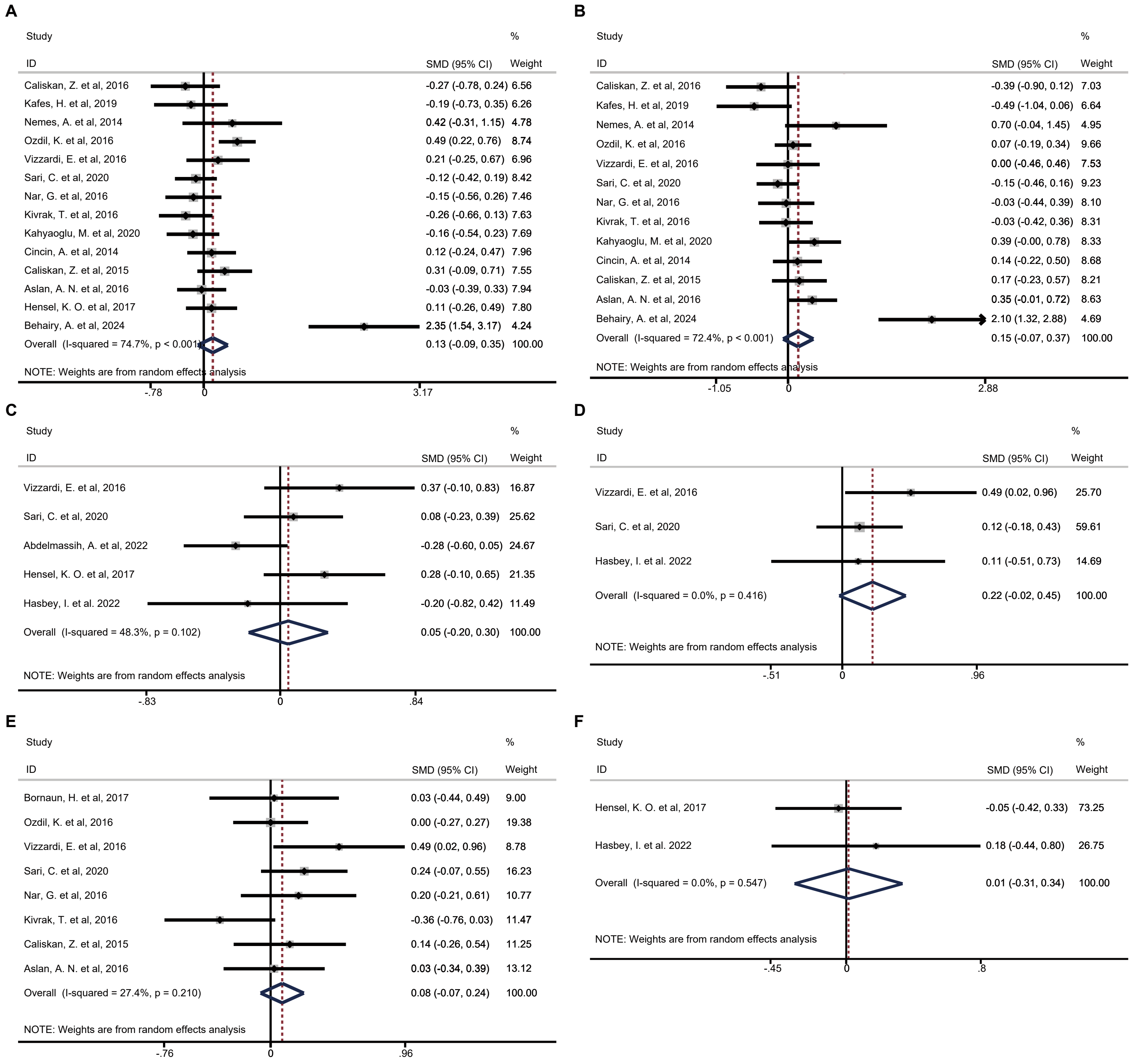

Figure S1. Forest plots comparing cardiac structure parameters between IBD patients and healthy controls.

(A) Left ventricular end-diastolic diameter. (B) Left ventricular end-systolic diameter. (C) Left ventricular end-diastolic volume. (D) Left ventricular end-systolic volume. (E) Left atrial diameter. (F) Left ventricular mass. IBD, inflammatory bowel disease. SMD, standardized mean difference. CI, confidence interval.

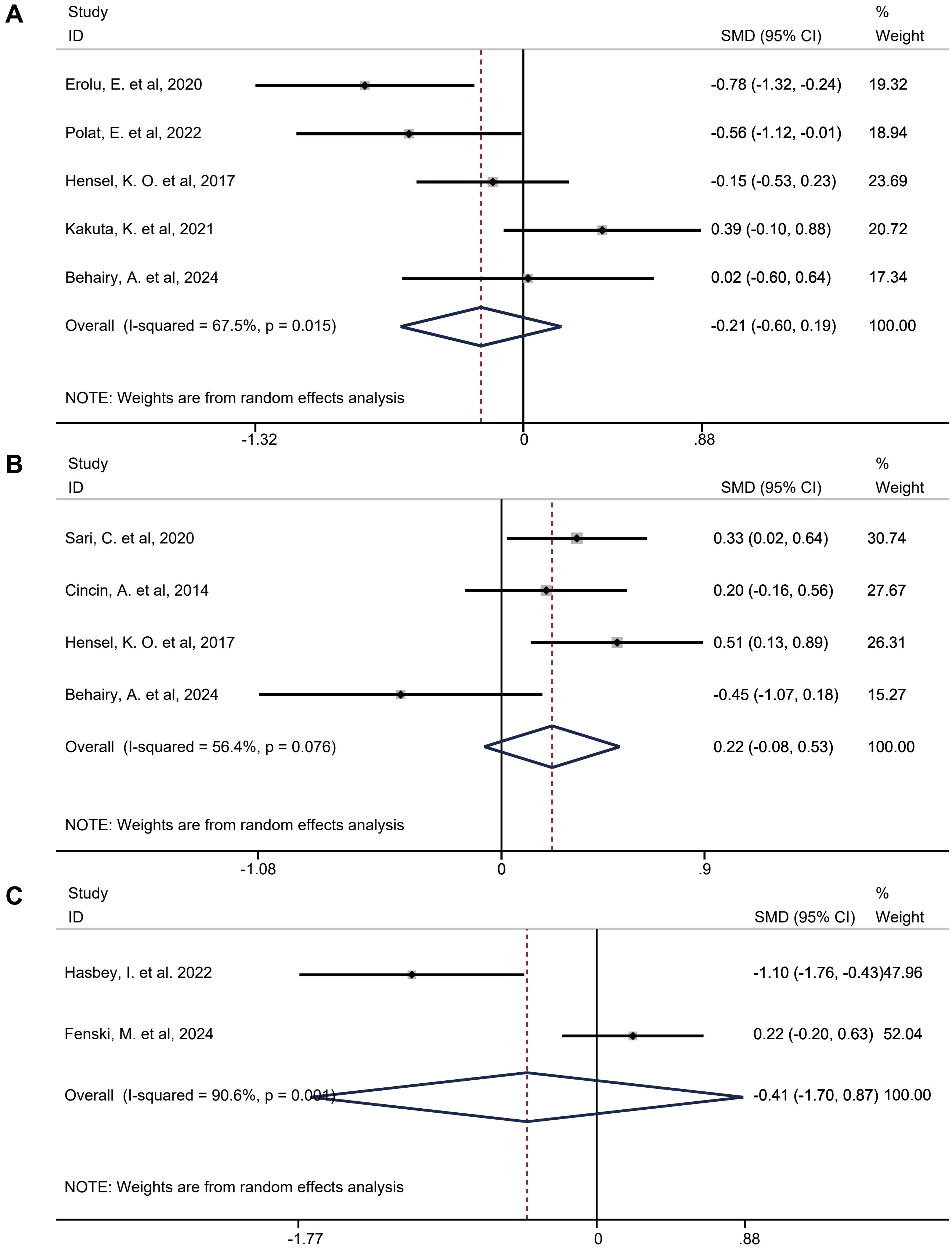

Figure S2. Forest plots comparing cardiac systolic parameters between IBD patients and healthy controls.
(A) Fractional shortening. (B) Global circumferential strain. (C) Right ventricular ejection fraction. IBD, inflammatory bowel disease. SMD, standardized mean difference. CI, confidence interval.

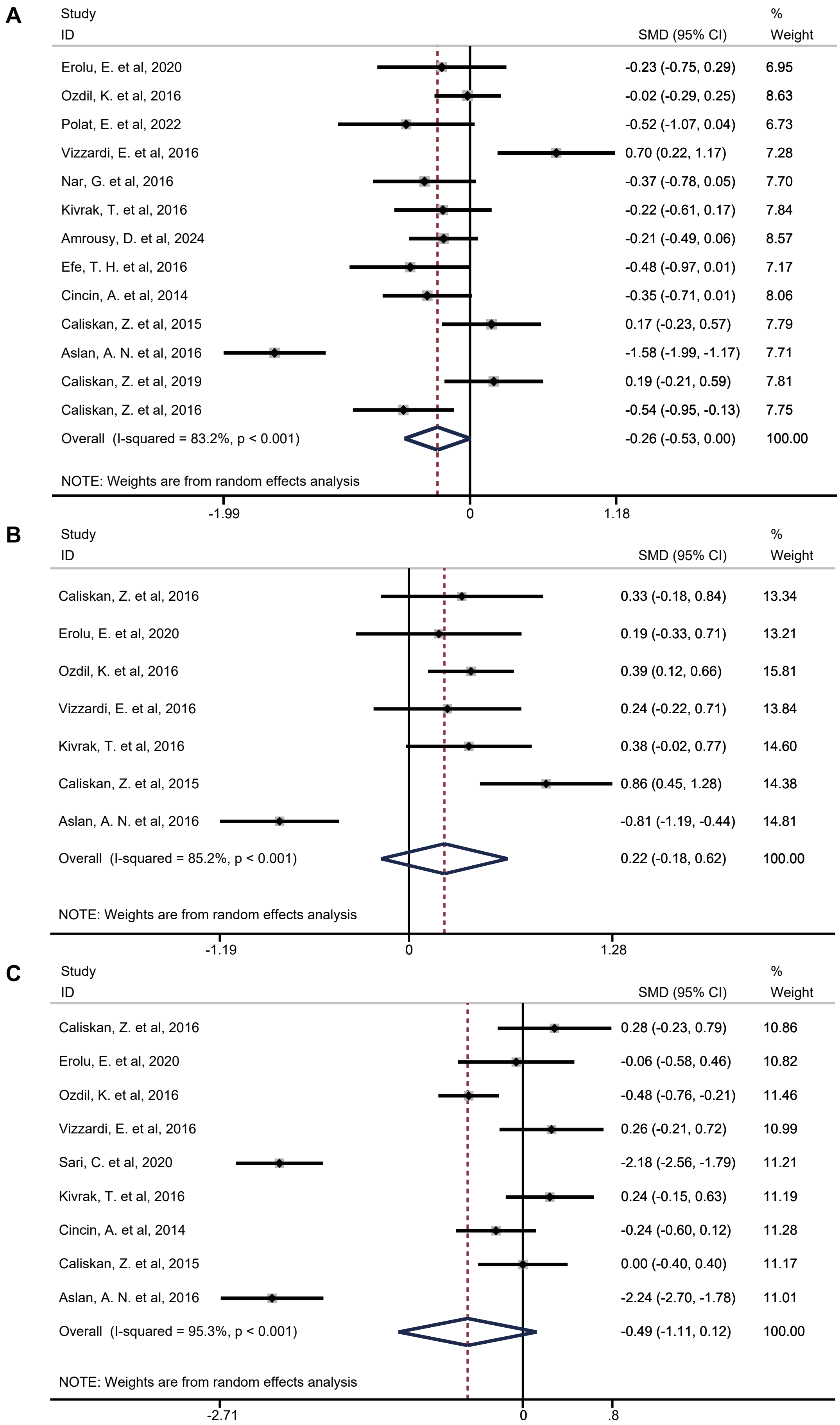

Figure S3. Forest plots comparing cardiac diastolic parameters between IBD patients and healthy controls.
(A) E wave. (B) A' wave. (C) E' wave. IBD, inflammatory bowel disease. SMD, standardized mean difference. CI, confidence interval.

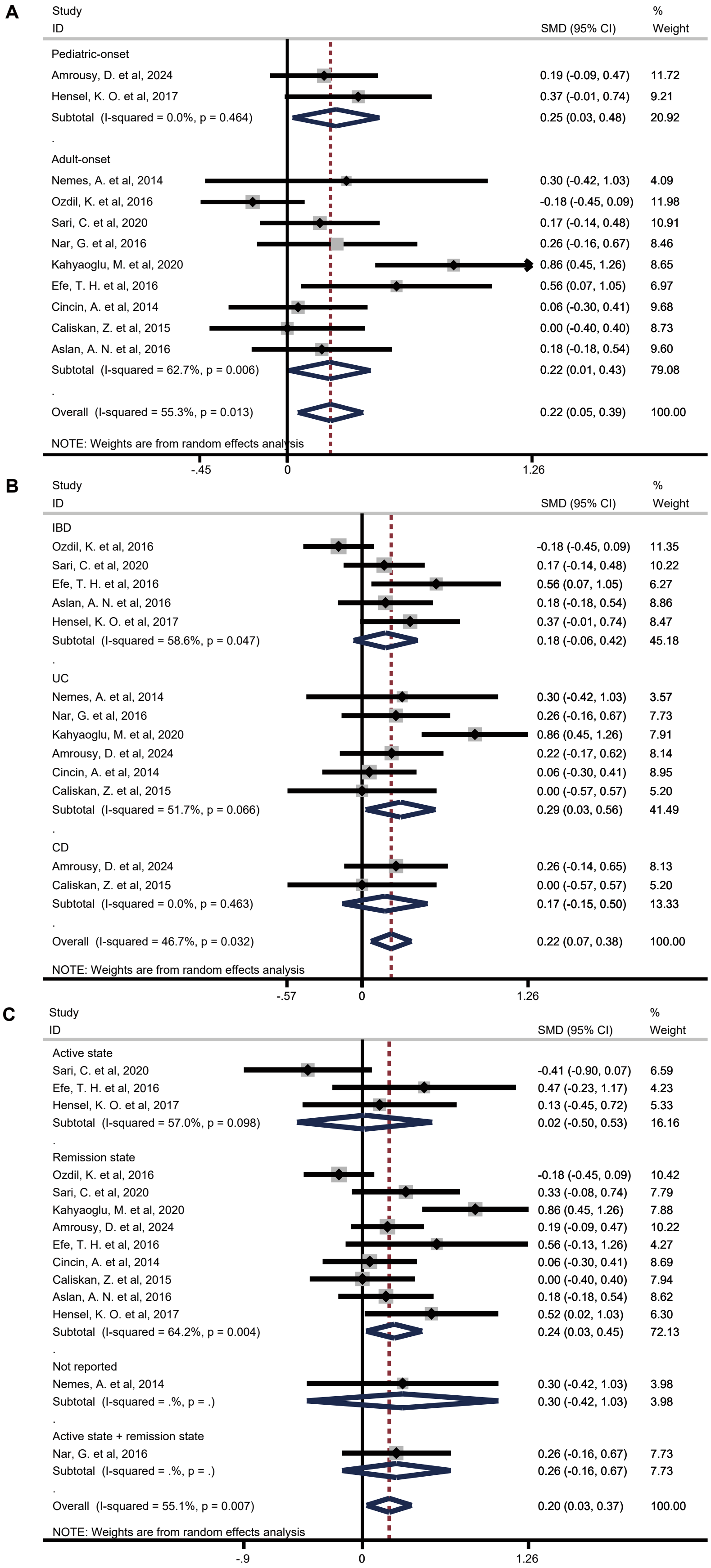

Figure S4. Subgroup analysis of interventricular septum between IBD patients and healthy controls.

(A) Age of onset. (B) IBD subtypes. (C) Disease activity. IBD, inflammatory bowel disease. SMD, standardized mean difference. CI, confidence interval. UC, ulcerative colitis. CD, Crohn's disease.

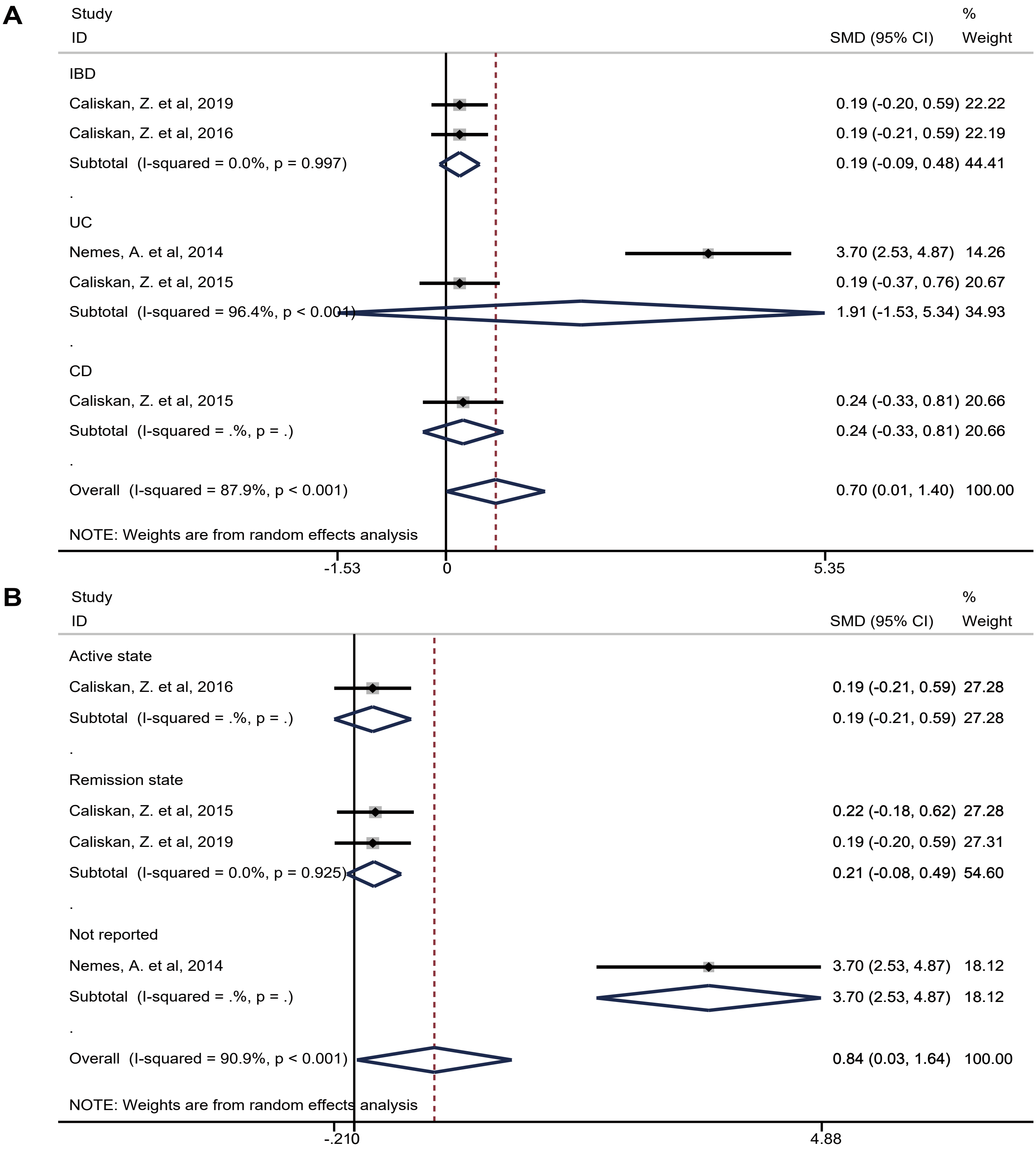

Figure S5. Subgroup analysis of left ventricular mass index between IBD patients and healthy controls.
(A) IBD subtypes. (B) Disease activity. IBD, inflammatory bowel disease. SMD, standardized mean difference. CI, confidence interval. UC, ulcerative colitis. CD, Crohn's disease.

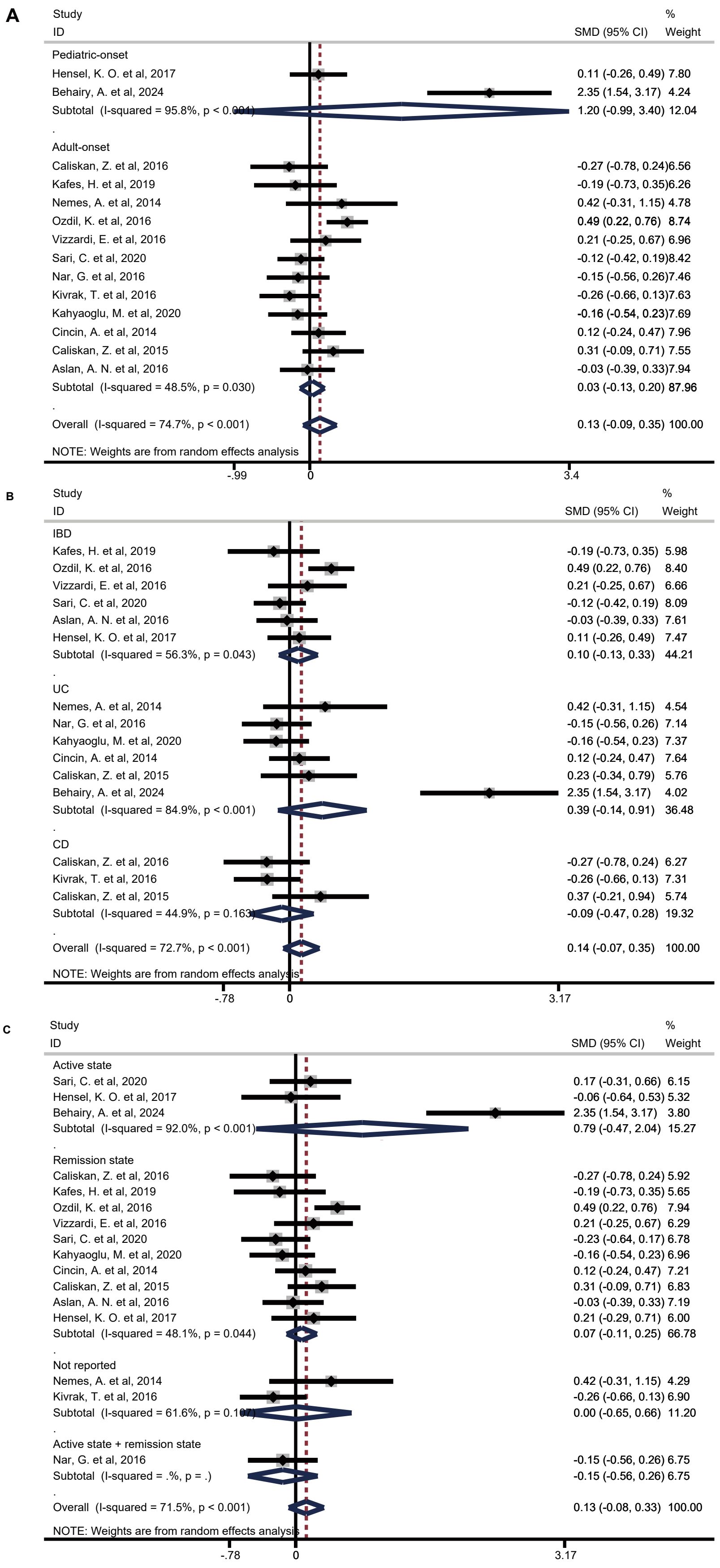

Figure S6. Subgroup analysis of left ventricular end-diastolic dimension between IBD patients and healthy controls.

(A) Age of onset. (B) IBD subtypes. (C) Disease activity. IBD, inflammatory bowel disease. SMD, standardized mean difference. CI, confidence interval. UC, ulcerative colitis. CD, Crohn's disease.

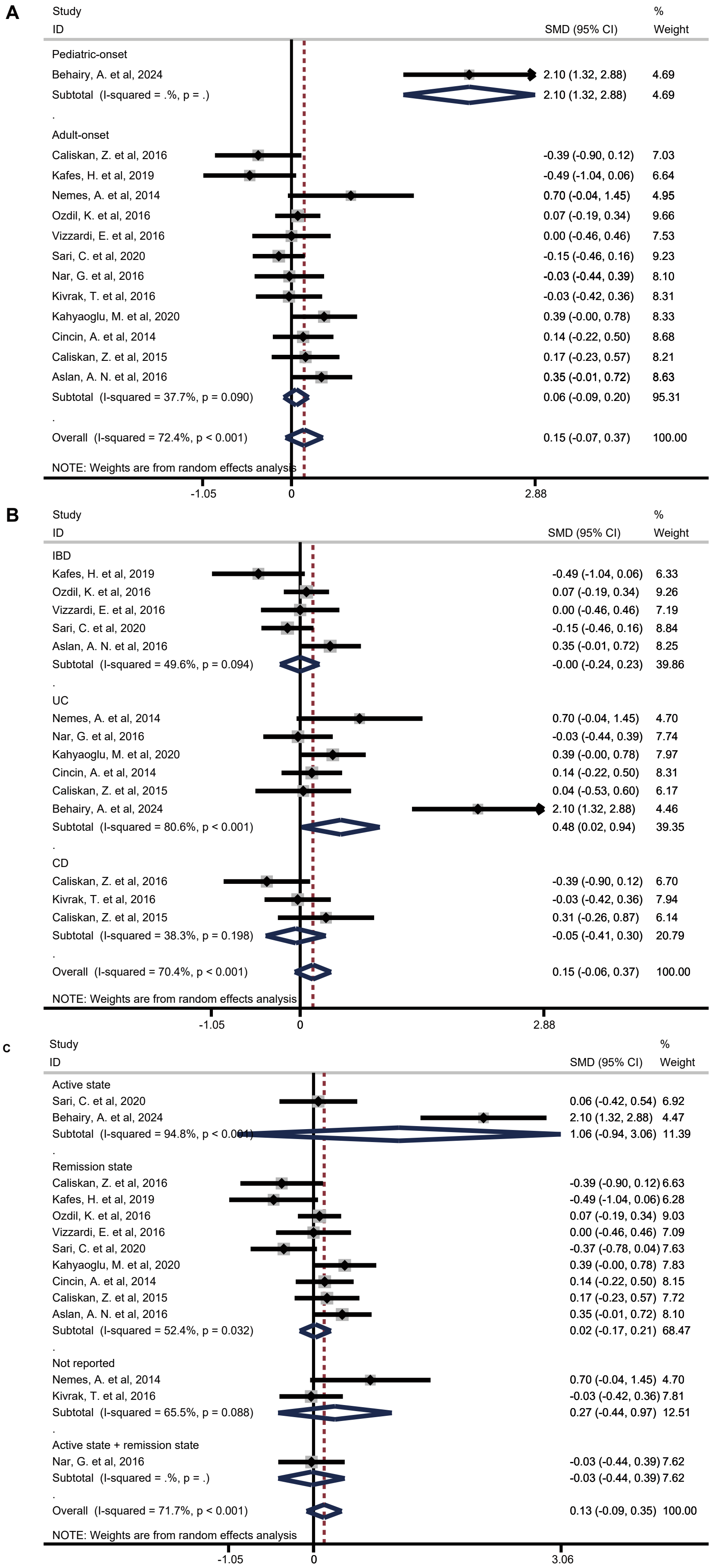

Figure S7. Subgroup analysis of left ventricular end-systolic dimension between IBD patients and healthy controls.

(A) Age of onset. (B) IBD subtypes. (C) Disease activity. IBD, inflammatory bowel disease. SMD, standardized mean difference. CI, confidence interval. UC, ulcerative colitis. CD, Crohn's disease.

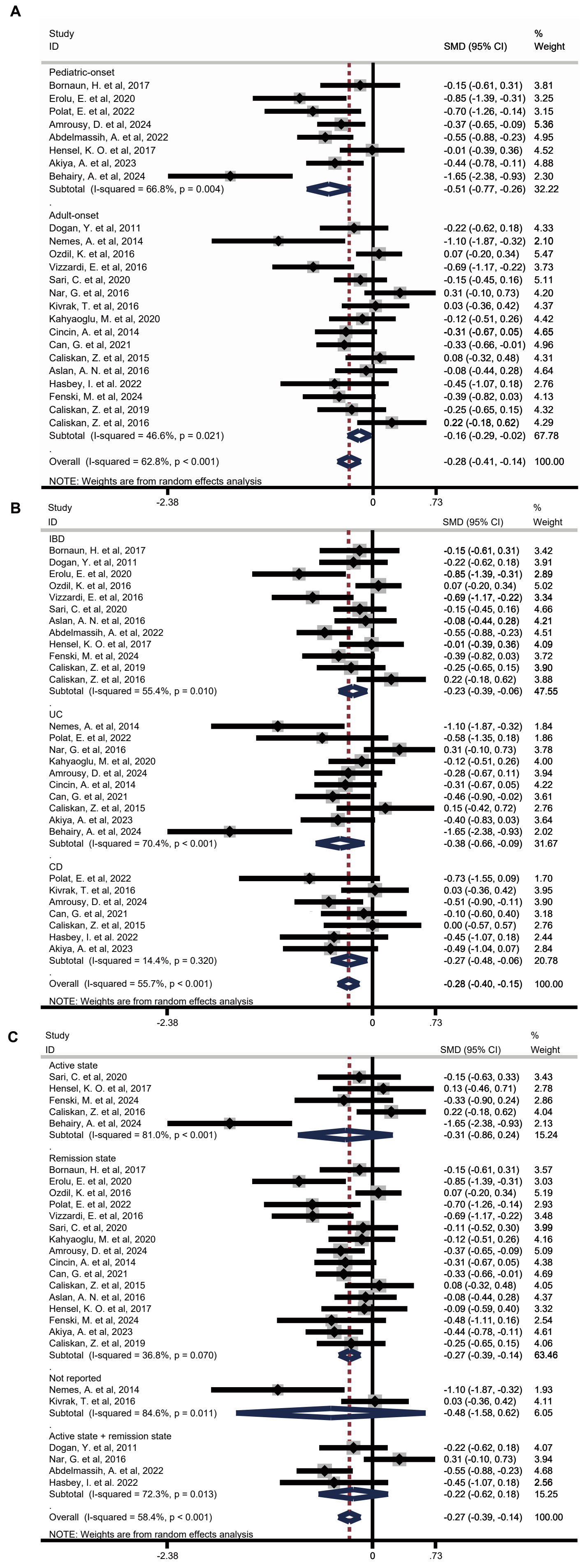

Figure S8. Subgroup analysis of ejection fraction between IBD patients and healthy controls. (A) Age of onset. (B) IBD subtypes. (C) Disease activity. IBD, inflammatory bowel disease. SMD, standardized mean difference. CI, confidence interval. UC, ulcerative colitis. CD, Crohn's disease.

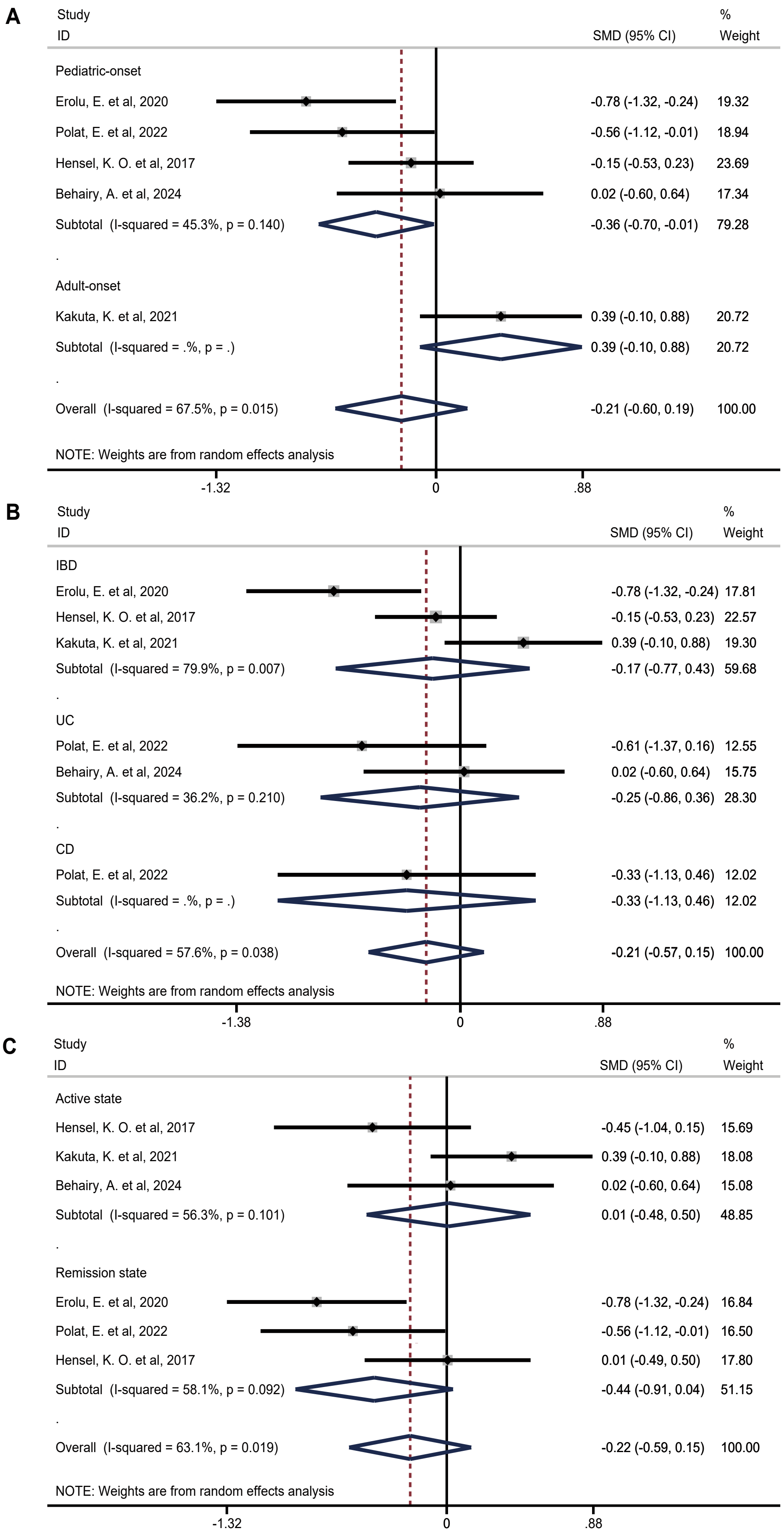

Figure S9. Subgroup analysis of fractional shortening between IBD patients and healthy controls. (A) Age of onset. (B) IBD subtypes. (C) Disease activity. IBD, inflammatory bowel disease. SMD, standardized mean difference. CI, confidence interval. UC, ulcerative colitis. CD, Crohn's disease.

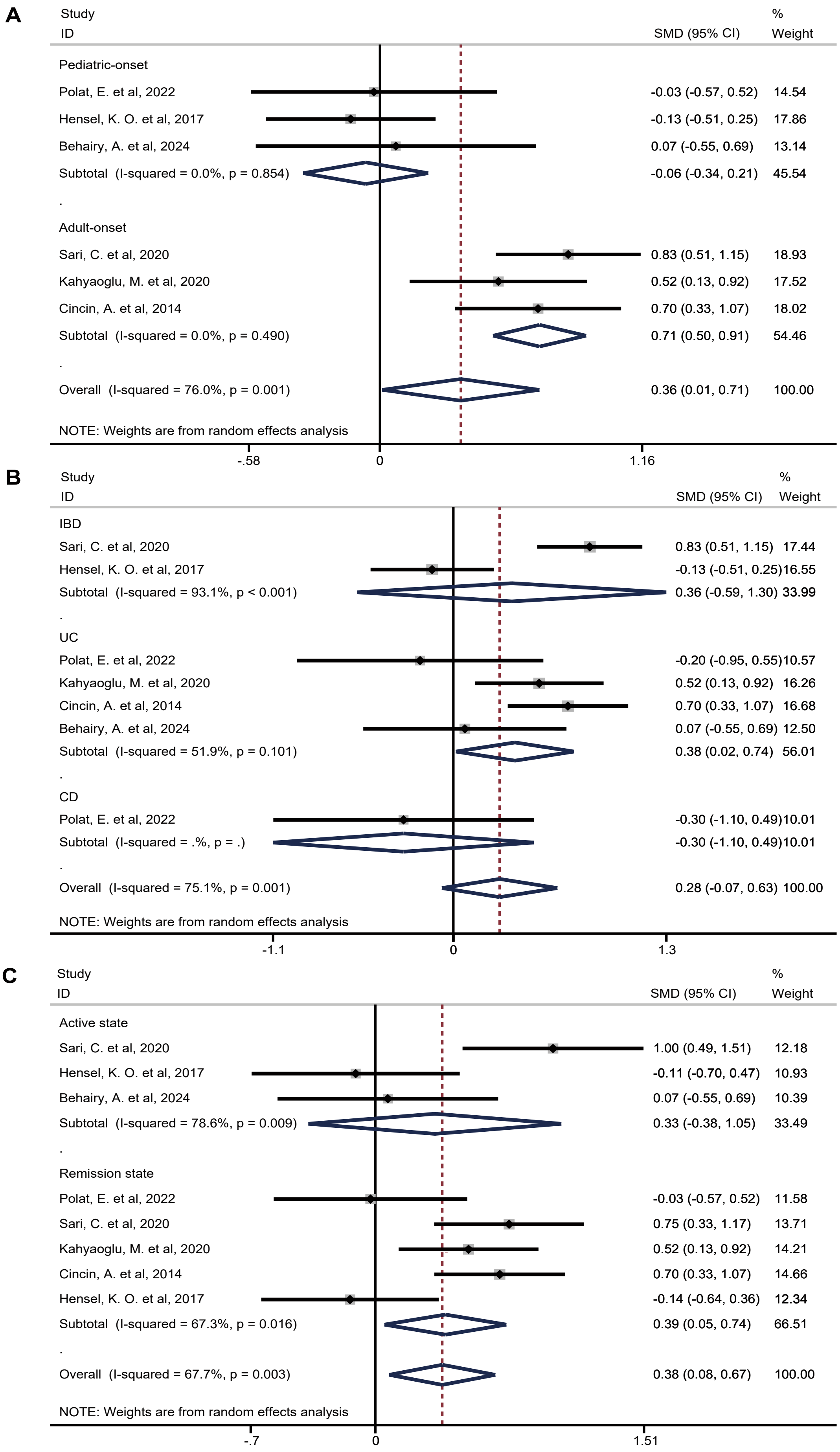

Figure S10. Subgroup analysis of global longitudinal strain between IBD patients and healthy controls.

(A) Age of onset. (B) IBD subtypes. (C) Disease activity. IBD, inflammatory bowel disease. SMD, standardized mean difference. CI, confidence interval. UC, ulcerative colitis. CD, Crohn's disease.

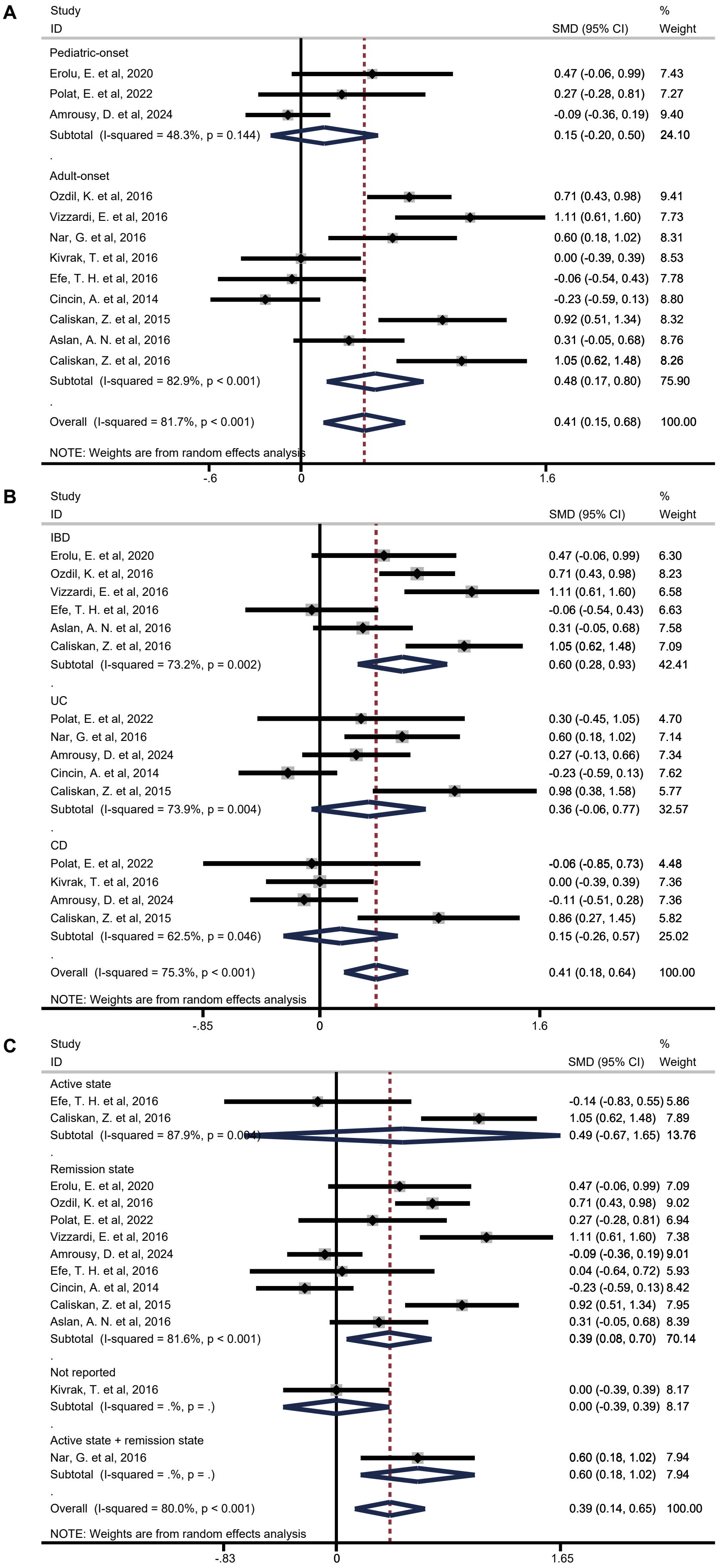

Figure S11. Subgroup analysis of A wave between IBD patients and healthy controls.
(A) Age of onset. (B) IBD subtypes. (C) Disease activity. IBD, inflammatory bowel disease. SMD, standardized mean difference. CI, confidence interval. UC, ulcerative colitis. CD, Crohn's disease.

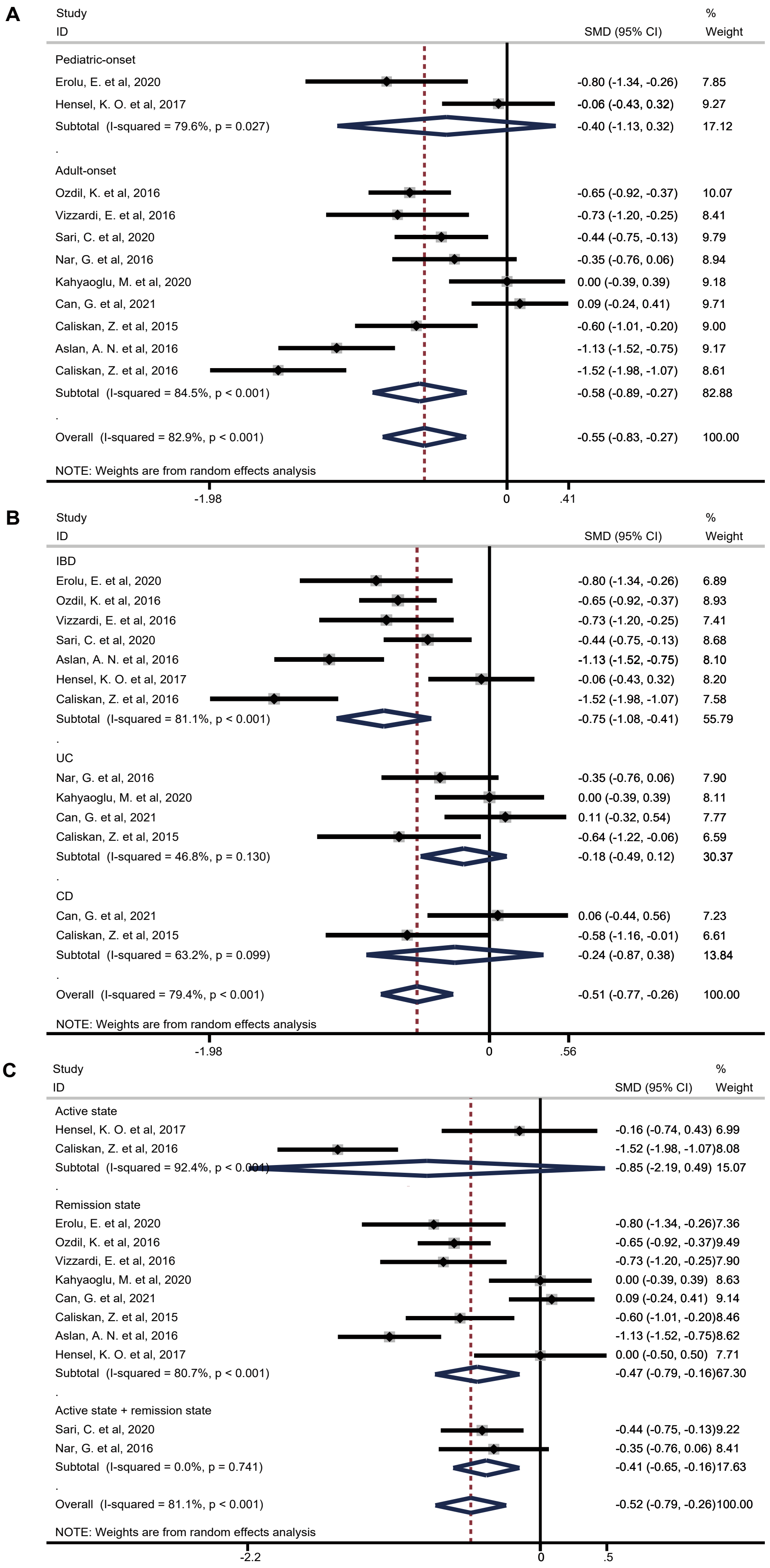

Figure S12. Subgroup analysis of E/A ratio between IBD patients and healthy controls.
(A) Age of onset. (B) IBD subtypes. (C) Disease activity. IBD, inflammatory bowel disease. SMD, standardized mean difference. CI, confidence interval. UC, ulcerative colitis. CD, Crohn's disease.

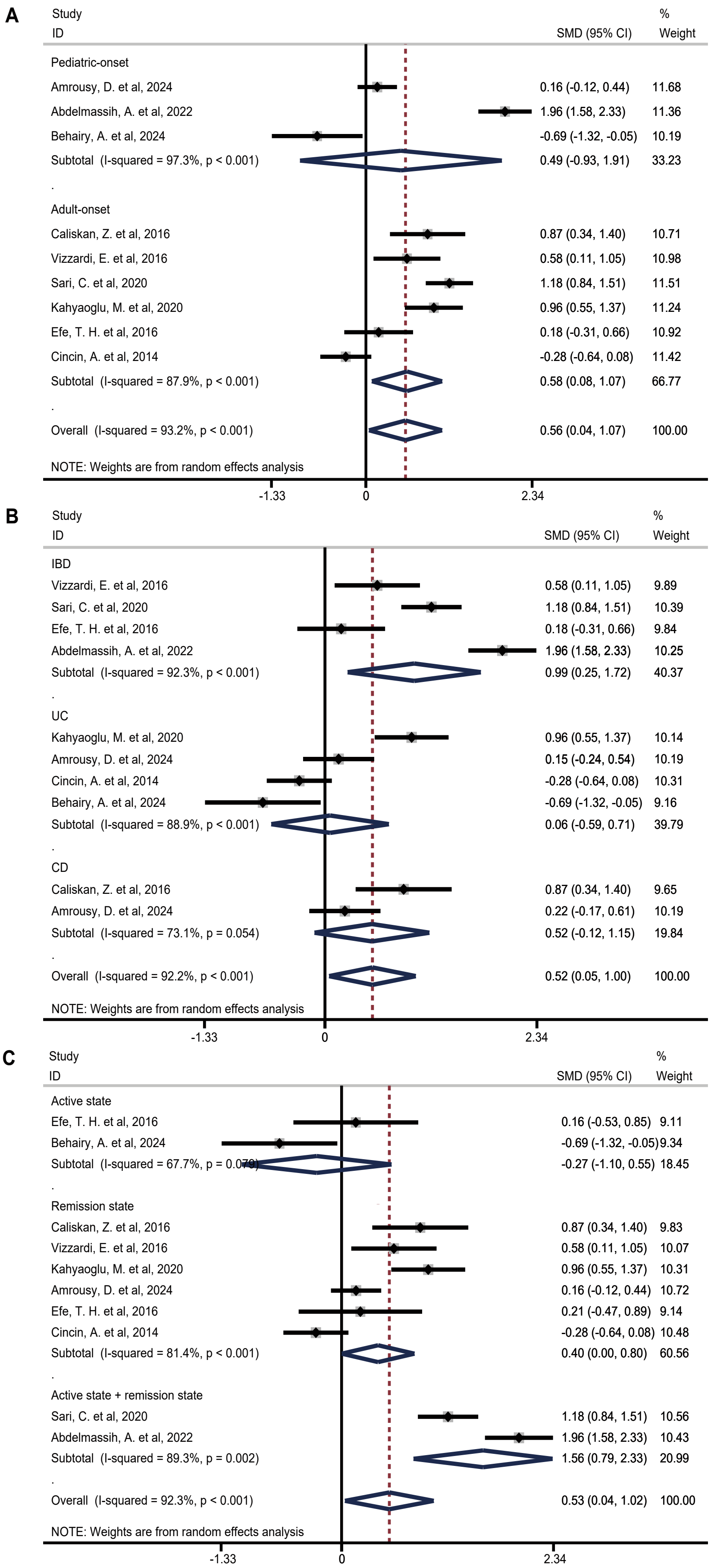

Figure S13. Subgroup analysis of E/E' ratio between IBD patients and healthy controls. (A) Age of onset. (B) IBD subtypes. (C) Disease activity. IBD, inflammatory bowel disease. SMD, standardized mean difference. CI, confidence interval. UC, ulcerative colitis. CD, Crohn's disease.

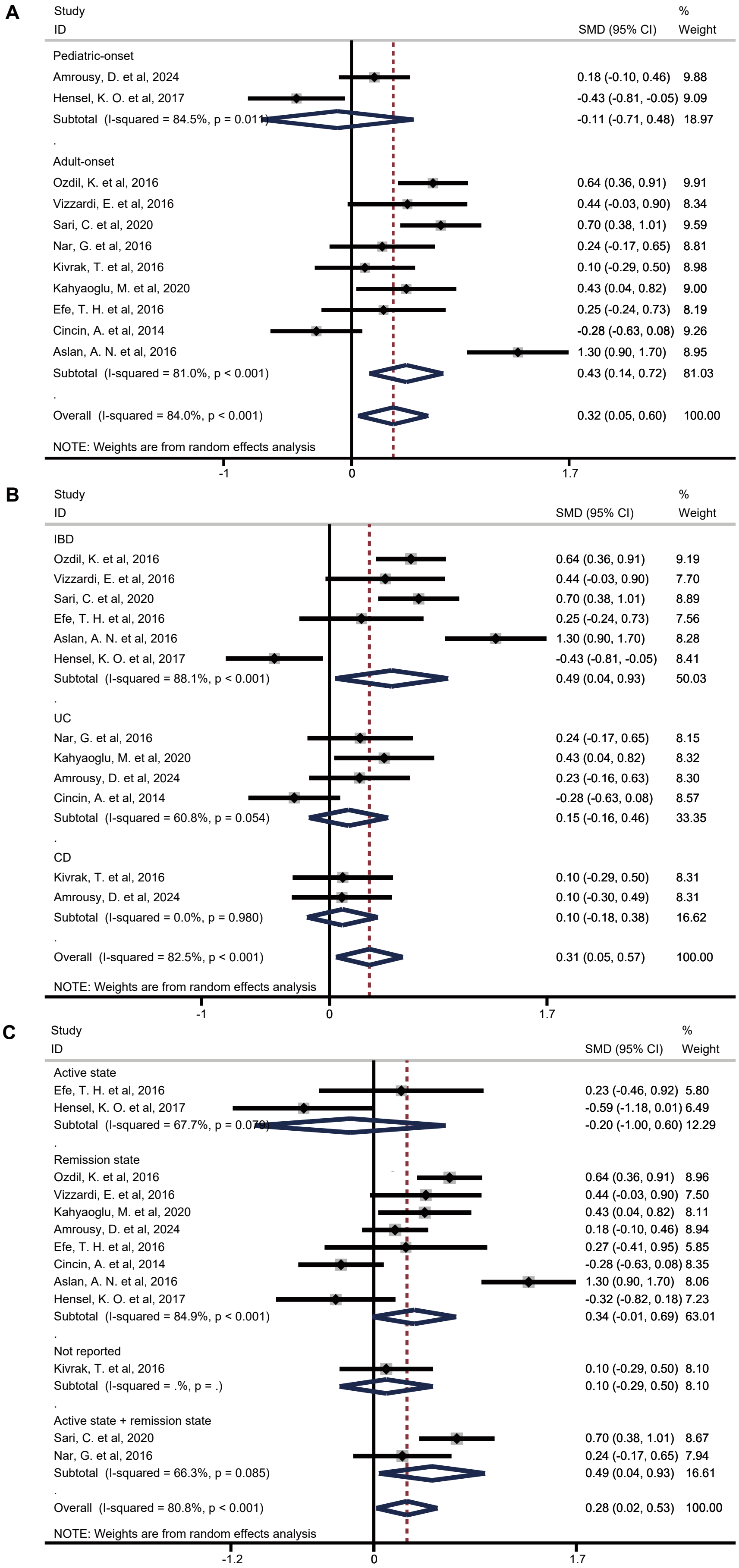

Figure S14. Subgroup analysis of mitral deceleration time between IBD patients and healthy controls.

(A) Age of onset. (B) IBD subtypes. (C) Disease activity. IBD, inflammatory bowel disease. SMD, standardized mean difference. CI, confidence interval. UC, ulcerative colitis. CD, Crohn's disease.

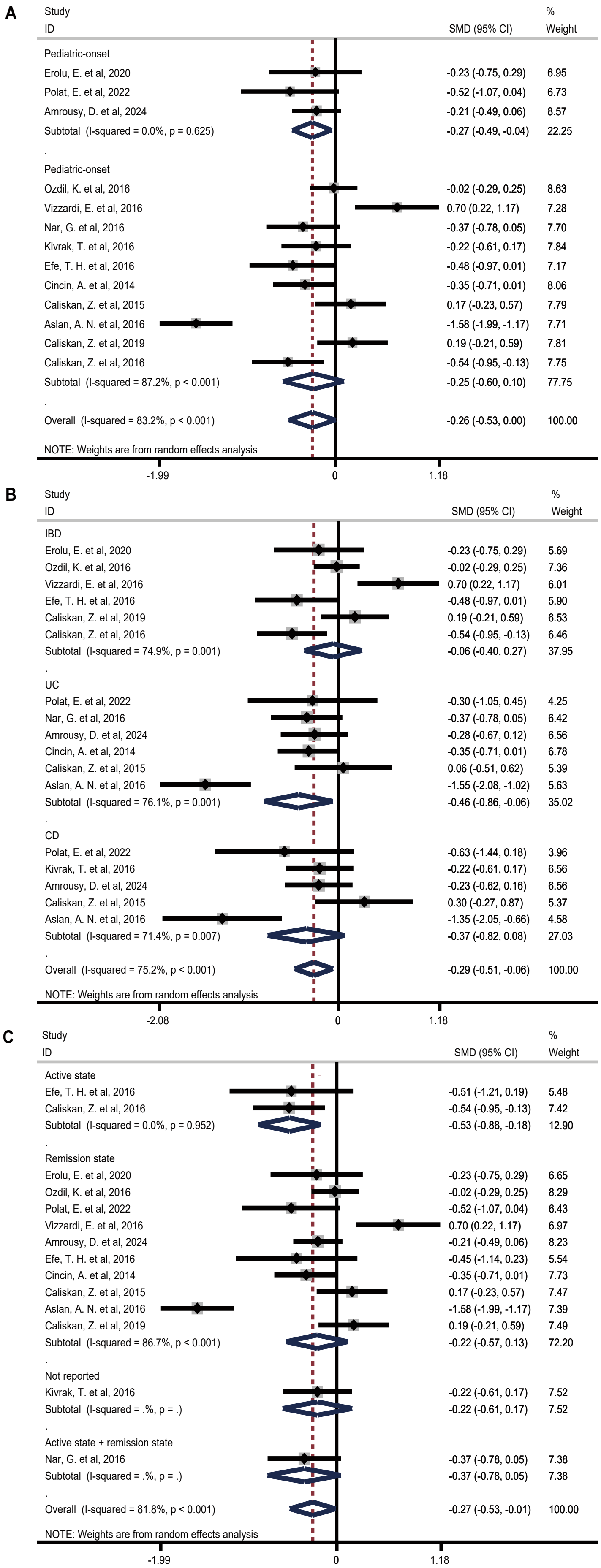

Figure S15. Subgroup analysis of E wave between IBD patients and healthy controls.
(A) Age of onset. (B) IBD subtypes. (C) Disease activity. IBD, inflammatory bowel disease. SMD, standardized mean difference. CI, confidence interval. UC, ulcerative colitis. CD, Crohn's disease.

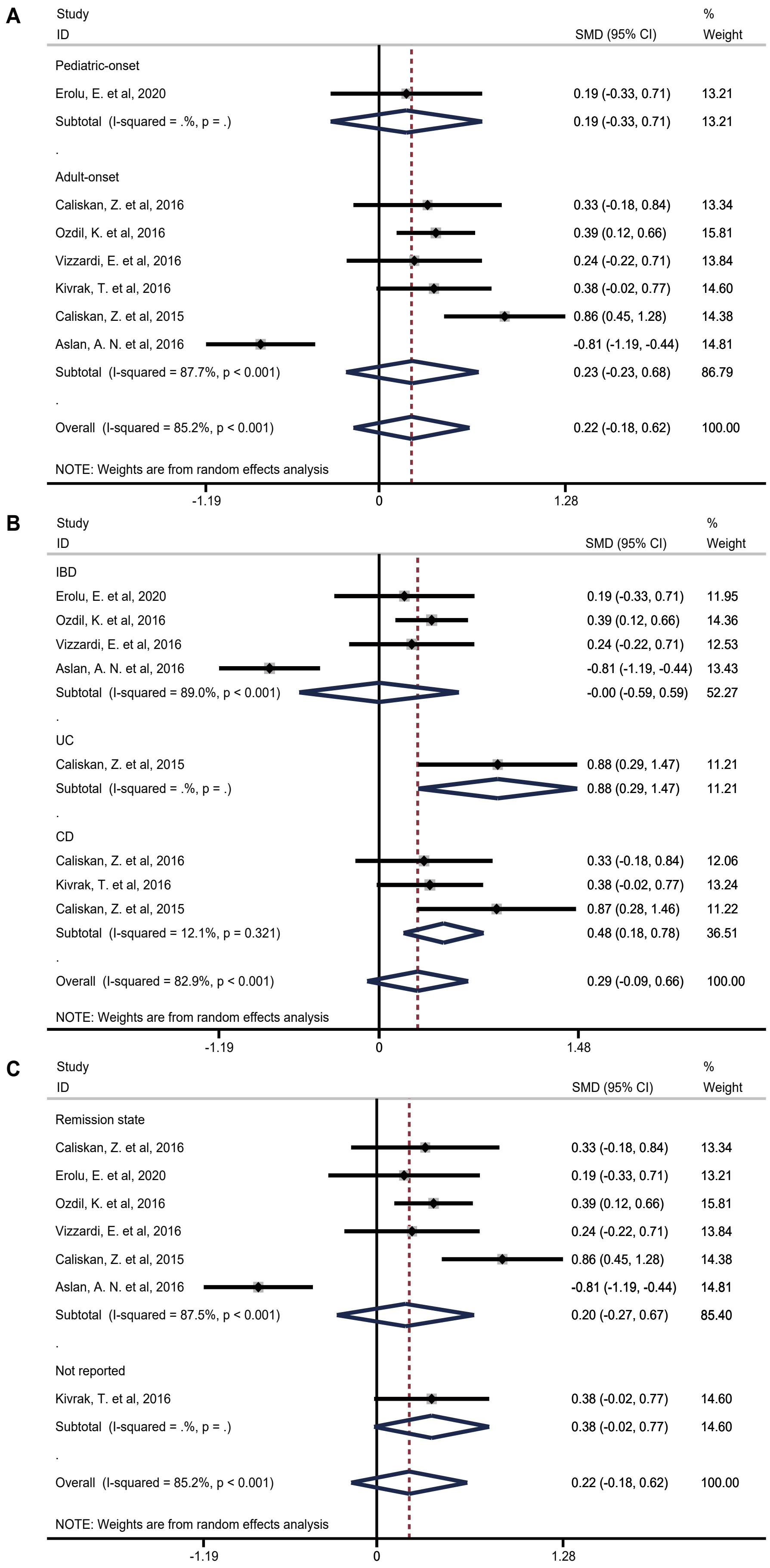

Figure S16. Subgroup analysis of A' wave between IBD patients and healthy controls.
(A) Age of onset. (B) IBD subtypes. (C) Disease activity. IBD, inflammatory bowel disease. SMD, standardized mean difference. CI, confidence interval. UC, ulcerative colitis. CD, Crohn's disease.

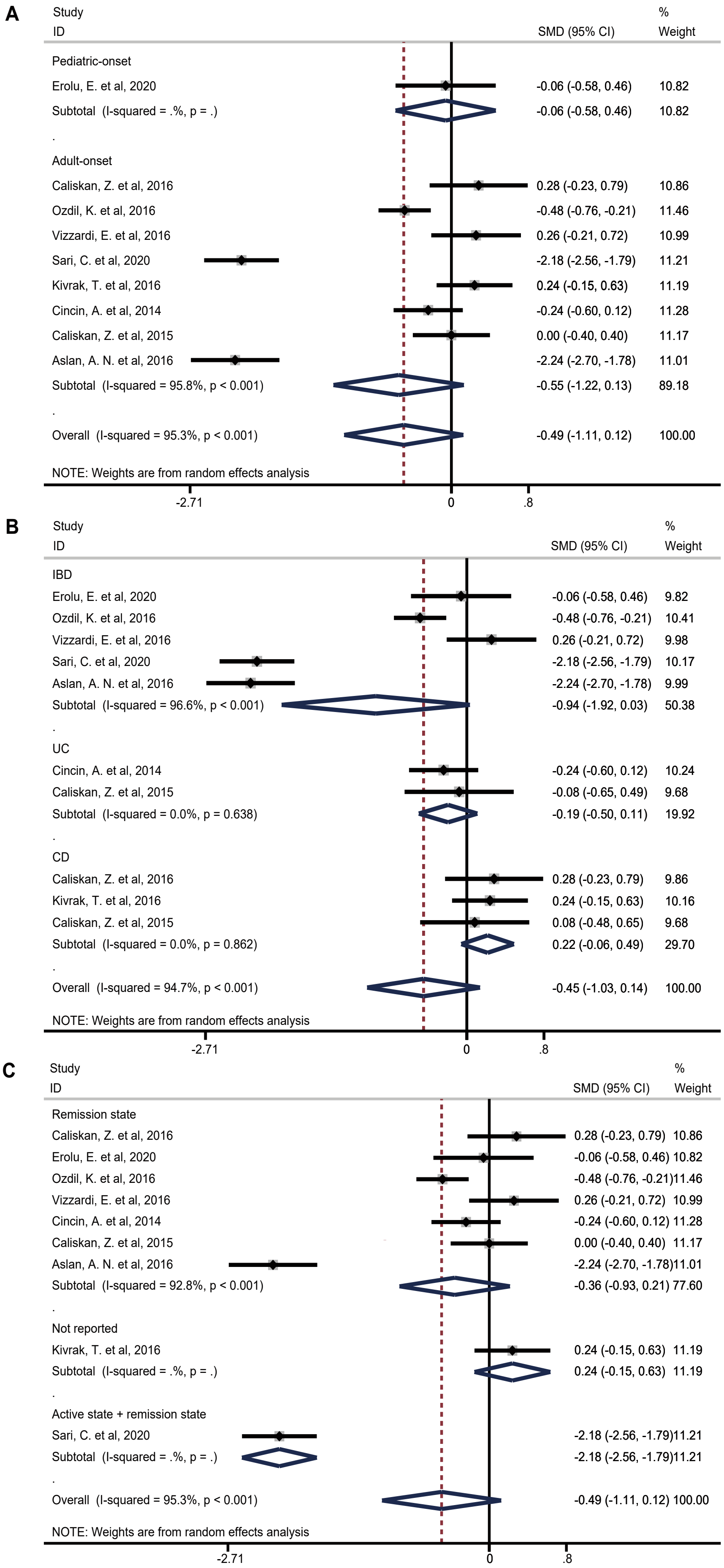

Figure S17. Subgroup analysis of E' wave between IBD patients and healthy controls.
(A) Age of onset. (B) IBD subtypes. (C) Disease activity. IBD, inflammatory bowel disease. SMD, standardized mean difference. CI, confidence interval. UC, ulcerative colitis. CD, Crohn's disease.

Table S1. PRISMA 2020 checklist of this study.

PRISMA, Preferred Reporting Items for Systematic Reviews and Meta-Analyses.

Section and Topic	Item #	Checklist item	Location where item is reported
**TITLE**			
Title	1	Identify the report as a systematic review.	Title
**ABSTRACT**			
Abstract	2	See the PRISMA 2020 for Abstracts checklist.	Abstract
**INTRODUCTION**			
Rationale	3	Describe the rationale for the review in the context of existing knowledge.	Introduction
Objectives	4	Provide an explicit statement of the objective(s) or question(s) the review addresses.	Introduction
**METHODS**			
Eligibility criteria	5	Specify the inclusion and exclusion criteria for the review and how studies were grouped for the syntheses.	Methods
Information sources	6	Specify all databases, registers, websites, organisations, reference lists and other sources searched or consulted to identify studies. Specify the date when each source was last searched or consulted.	Methods
Search strategy	7	Present the full search strategies for all databases, registers and websites, including any filters and limits used.	Methods
Selection process	8	Specify the methods used to decide whether a study met the inclusion criteria of the review, including how many reviewers screened each record and each report retrieved, whether they worked independently, and if applicable, details of automation tools used in the process.	Methods
Data collection process	9	Specify the methods used to collect data from reports, including how many reviewers collected data from each report, whether they worked independently, any processes for obtaining or confirming data from study investigators, and if applicable, details of automation tools used in the process.	Methods
Data items	10a	List and define all outcomes for which data were sought. Specify whether all results that were compatible with each outcome domain in each study were sought (e.g. for all measures, time points, analyses), and if not, the methods used to decide which results to collect.	Methods
	10b	List and define all other variables for which data were sought (e.g. participant and intervention characteristics, funding sources). Describe any assumptions made about any missing or unclear information.	Methods
Study risk of bias assessment	11	Specify the methods used to assess risk of bias in the included studies, including details of the tool(s) used, how many reviewers assessed each study and whether they worked independently, and if applicable, details of automation tools used in the process.	Methods
Effect measures	12	Specify for each outcome the effect measure(s) (e.g. risk ratio, mean difference) used in the synthesis or presentation of results.	Methods
Synthesis methods	13a	Describe the processes used to decide which studies were eligible for each synthesis (e.g. tabulating the study intervention characteristics and comparing against the planned groups for each synthesis (item #5)).	Methods
	13b	Describe any methods required to prepare the data for presentation or synthesis, such as handling of missing summary statistics, or data conversions.	Methods
	13c	Describe any methods used to tabulate or visually display results of individual studies and syntheses.	Methods
	13d	Describe any methods used to synthesize results and provide a rationale for the choice(s). If meta-analysis was performed, describe the model(s), method(s) to identify the presence and extent of statistical heterogeneity, and software package(s) used.	Methods
	13e	Describe any methods used to explore possible causes of heterogeneity among study results (e.g. subgroup analysis, metaregression).	Methods
	13f	Describe any sensitivity analyses conducted to assess robustness of the synthesized results.	Methods
Reporting bias assessment	14	Describe any methods used to assess risk of bias due to missing results in a synthesis (arising from reporting biases).	Methods
Certainty assessment	15	Describe any methods used to assess certainty (or confidence) in the body of evidence for an outcome.	Methods
**RESULTS**			
Study selection	16a	Describe the results of the search and selection process, from the number of records identified in the search to the number of studies included in the review, ideally using a flow diagram.	Methods/Results
	16b	Cite studies that might appear to meet the inclusion criteria, but which were excluded, and explain why they were excluded.	Methods/Results
Study characteristics	17	Cite each included study and present its characteristics.	Results
Risk of bias in studies	18	Present assessments of risk of bias for each included study.	Results
Results of individual studies	19	For all outcomes, present, for each study: (a) summary statistics for each group (where appropriate) and (b) an effect estimate and its precision (e.g. confidence/credible interval), ideally using structured tables or plots.	Results
Results of syntheses	20a	For each synthesis, briefly summarise the characteristics and risk of bias among contributing studies.	Results
	20b	Present results of all statistical syntheses conducted. If meta-analysis was done, present for each the summary estimate and its precision (e.g. confidence/credible interval) and measures of statistical heterogeneity. If comparing groups, describe the direction of the effect.	Results
	20c	Present results of all investigations of possible causes of heterogeneity among study results.	Results
	20d	Present results of all sensitivity analyses conducted to assess the robustness of the synthesized results.	Results
Reporting biases	21	Present assessments of risk of bias due to missing results (arising from reporting biases) for each synthesis assessed.	Results
Certainty of evidence	22	Present assessments of certainty (or confidence) in the body of evidence for each outcome assessed.	Results
**DISCUSSION**			
Discussion	23a	Provide a general interpretation of the results in the context of other evidence.	Discussion
	23b	Discuss any limitations of the evidence included in the review.	Discussion/Limitations
	23c	Discuss any limitations of the review processes used.	Discussion/Limitations
	23d	Discuss implications of the results for practice, policy, and future research.	Discussion/Limitations
**OTHER INFORMATION**			
Registration and protocol	24a	Provide registration information for the review, including register name and registration number, or state that the review was not registered.	Methods
	24b	Indicate where the review protocol can be accessed, or state that a protocol was not prepared.	Methods
	24c	Describe and explain any amendments to information provided at registration or in the protocol.	Methods, Supplementary information
Support	25	Describe sources of financial or non-financial support for the review, and the role of the funders or sponsors in the review.	Acknowledgments
Competing interests	26	Declare any competing interests of review authors.	Declaration of interest
Availability of data, code and other materials	27	Report which of the following are publicly available and where they can be found: template data collection forms; data extracted from included studies; data used for all analyses; analytic code; any other materials used in the review.	Methods/Results

From: Page MJ, McKenzie JE, Bossuyt PM, Boutron I, Hoffmann TC, Mulrow CD, et al. The PRISMA 2020 statement: an updated guideline for reporting systematic reviews. BMJ 2021;372:n71. doi: 10.1136/bmj.n71. This work is licensed under CC BY 4.0. To view a copy of this license, visit https://creativecommons.org/licenses/by/4.0/

Data S1. Chinese database search strategy and screening results.

Databases: China National Knowledge Infrastructure
Date searched: 9 May 2026
Records retrieved: n = 3590
Eligible included: n = 0
Database search query：(SU%= '炎症性肠病' OR TKA= ('克罗恩病' + '溃疡性结肠炎' + '克罗恩性肠炎' + '局限性肠炎' + '回结肠炎' + '末端回肠炎' + '局限性回肠炎' + '肉芽肿性肠炎' + '肉芽肿性结肠炎' + '重症结肠炎' + '特发性直肠结肠炎' + 'IBD' + 'CD' + 'UC') OR KY= ('克罗恩病' + '溃疡性结肠炎' + '克罗恩性肠炎' + '局限性肠炎' + '回结肠炎' + '末端回肠炎' + '局限性回肠炎' + '肉芽肿性肠炎' + '肉芽肿性结肠炎' + '重症结肠炎' + '特发性直肠结肠炎' + 'IBD' + 'CD' + 'UC')) AND (SU%= '超声心动图' + '心超' OR TKA='心脏超声检查' + '心脏扫描' + '心肌扫描' + '心脏磁共振' + '心功能' + '心脏结构' + '收缩功能' + '舒张功能' + '心室' + '室间隔' + '每搏输出量' + '射血分数' + '短轴缩短率' + 'E/A比值' + '整体纵向应变' + '圆周应变' + '超声心动图' + '心超' OR KY='心脏超声检查' + '心脏扫描' + '心肌扫描' + '心脏磁共振' + '心功能' + '心脏结构' + '收缩功能' + '舒张功能' + '心室' + '室间隔' + '每搏输出量' + '射血分数' + '短轴缩短率' + 'E/A比值' + '整体纵向应变' + '圆周应变' + '超声心动图' + '心超') NOT (TKA='病例报告' + '病例研究' + '病例对照研究' + '社论' + '历史文献' + '评论' + '信件' + '通讯' + '啮齿目' + '啮齿动物' + '实验动物' + '动物实验' + '动物模型' + '动物建模' + '非人类')

Databases: Wanfang Med Online
Date searched: 9 May 2026
Records retrieved: n = 3113
Eligible included: n = 0
Database search query：((主题=炎症性肠病) OR (题名或关键词=(克罗恩病 OR 溃疡性结肠炎 OR 克罗恩性肠炎 OR 局限性肠炎 OR 回结肠炎 OR 末端回肠炎 OR 局限性回肠炎 OR 肉芽肿性肠炎 OR 肉芽肿性结肠炎 OR 重症结肠炎 OR 特发性直肠结肠炎 OR IBD OR CD OR UC))) AND ((主题=(超声心动图 OR 心超)) OR (题名或关键词=(心脏超声检查 OR 心脏扫描 OR 心肌扫描 OR 心脏磁共振 OR 心功能 OR 心脏结构 OR 收缩功能 OR 舒张功能 OR 心室 OR 室间隔 OR 每搏输出量 OR 射血分数 OR 短轴缩短率 OR E/A比值 OR 整体纵向应变 OR 圆周应变 OR 超声心动图 OR 心超))) NOT (题名或关键词=(病例报告 OR 病例研究 OR 病例对照研究 OR 社论 OR 历史文献 OR 评论 OR 信件 OR 通讯 OR 啮齿目 OR 啮齿动物 OR 实验动物 OR 动物实验 OR 动物模型 OR 动物建模 OR 非人类))
